# Mass Sportrometry: An annual look back at applications of mass spectrometry in sport and exercise science

**DOI:** 10.1002/ansa.202300003

**Published:** 2023-03-31

**Authors:** Marilyn LY Ong, Christopher G Green, Samantha N Rowland, Liam M Heaney

**Affiliations:** ^1^ School of Sport Exercise and Health Sciences Loughborough University Loughborough UK; ^2^ School of Health Sciences Exercise and Sports Science Programme Universiti Sains Malaysia Kota Bharu Malaysia

**Keywords:** analytical science, exercise, mass spectrometry, nutrition, sport

## Abstract

Research in sport and exercise science (SES) is reliant on robust analyses of biomarker measurements to assist with the interpretation of physiological outcomes. Mass spectrometry (MS) is an analytical approach capable of highly sensitive, specific, precise, and accurate analyses of a range of biomolecules, many of which are of interest in SES including, but not limited to, endogenous metabolites, exogenously administered compounds (e.g. supplements), mineral ions, and circulating/tissue proteins. This annual review provides a summary of the applications of MS across studies investigating aspects related to sport or exercise in manuscripts published, or currently in press, in 2022. In total, 93 publications are included and categorized according to their methodologies including targeted analyses, metabolomics, lipidomics, proteomics, and isotope ratio/elemental MS. The advantageous analytical opportunities afforded by MS technologies are discussed across a selection of relevant articles. In addition, considerations for the future of MS in SES, including the need to improve the reporting of assay characteristics and validation data, are discussed, alongside the recommendation for selected current methods to be superseded by MS‐based approaches where appropriate. The review identifies that a targeted, mostly quantitative, approach is the most commonly applied MS approach within SES, although there has also been a keen interest in the use of *‘omics’* to perform hypothesis‐generating research studies. Nonetheless, MS is not commonplace in SES at this time, but its use to expand, and possibly improve, the analytical options should be continually considered to exploit the benefits of analytical chemistry in exercise/sports‐based research. Overall, it is exciting to see the gradually increasing adoption of MS in SES and it is expected that the number, and quality, of MS‐based assays in SES will increase over time, with the potential for 2023 to further establish this technique within the field.

**Abbreviations**
ACSM: American College of Sports Medicine
AKI: Acute kidney injury
ATP: Adenosine triphosphate
CBD: cannabidiol
CHO: carbohydrate
Da: Dalton
DNA: deoxyribonucleic acid
eCBome: Endocannbinoidome
Ex Ket: Ketone monoester
FAHFAs: Fatty acid esters of hydroxy fatty acids
FBP: Fava bean protein
FDR: False discovery rate
FSH: Follicle‐stimulating hormone
FSR: Fractional synthesis rate
GC: Gas chromatography
ICP: Inductively coupled plasma
IL‐6: Interleukin‐6
IR: Isotope ratio
Lac‐Phe: N‐lactoylphenylalanine
Lac‐Val: N‐lactoylvaline
LC: Liquid chromatography
LH: Luteinizing hormone
m/z: mass‐to‐charge ratio
MS: Mass spectrometry
NPY: Neuropeptide Y
NWT: Normal weight trained
OPLS‐DA: Orthogonal partial least squares‐discriminant analysis
OWT: Overweight trained
PCA: Principal components analysis
PKPD: Pharmacokinetics/pharmacodynamics
SCFAs: Short‐chain fatty acids
SES: Sport and exercise science
T2DM: Type II diabetes mellitus
VIP: Variable importance in projection
VO2peak: Peak oxygen uptake
VT1: Ventilatory threshold‐1
WADA: World Anti‐Doping Agency

## 1 INTRODUCTION

**FIGURE 1:**
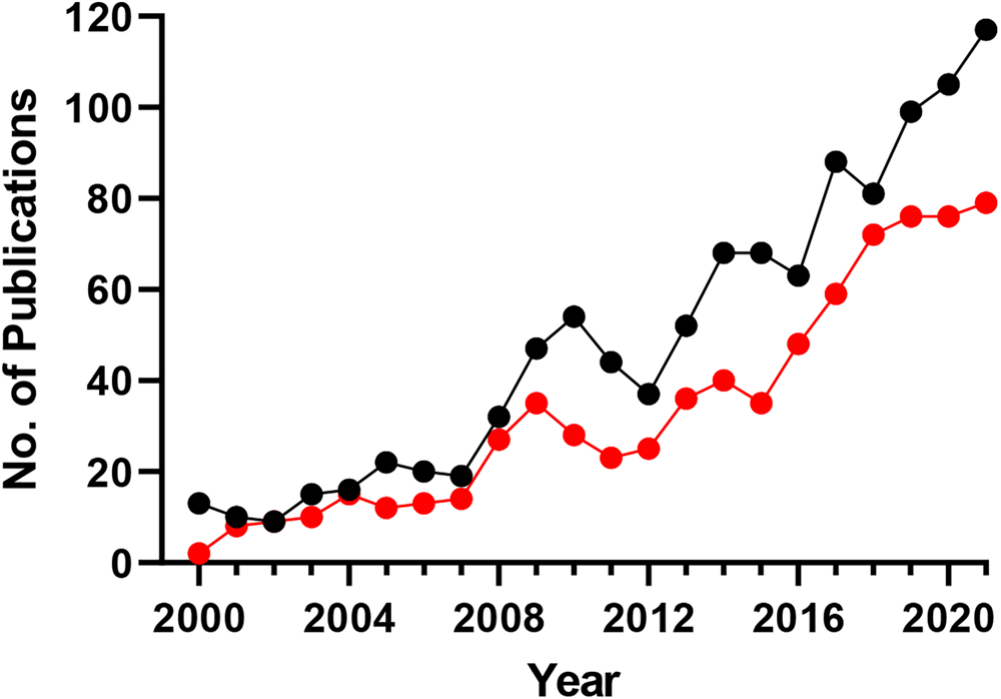
The total number of publications included via a PubMed search using the terms ‘mass spectrometry’ AND ‘sport OR exercise’ in the title and/or abstract (black markers) and the number of publications after further removal of those including the terms ‘doping OR anti‐doping OR drug OR drugs’ (red markers).

Research in sport and exercise science (SES) is inherently reliant on robust and reliable analyses of biomarker measurements. This is essential to aid the interpretation of physiological outcomes of the state/intervention being investigated. These measurements vary across exhaled respiratory gases (i.e. O_2_ and CO_2_ for oxygen consumption data), rapid metabolic readbacks (e.g. ear‐prick lactate levels), clinical chemistry analyses (e.g. inflammatory markers), as well as more bespoke approaches using a variety of advanced instrumentation. One of these advanced approaches, which has gradually and increasingly been adopted into SES over the previous two decades, is the use of mass spectrometry (MS) with a nine‐fold increase in mentions (title or abstract) in research manuscripts in 2021 compared to those in 2000. This increase remains even after the publication search is further filtered to remove those related to anti‐doping, an area where the use of MS is a mainstay in the modern era (Figure 1). The application of MS into SES research is an area that we, as a lab, have jovially coined *Mass Sportrometry*.

MS is a member of the field of analytical chemistry and is used to measure the relative molecular mass of a molecule which is in a charged state (i.e. an ion), termed as its mass‐to‐charge ratio (*m/z*).^1^ MS technologies provide the researcher with a capacity to measure, qualitatively and quantitatively, across a varied set of molecules ranging from small metabolites and peptides to larger biomolecular protein structures. This varied set of analytical targets, alongside the capacity to perform measurements with high levels of sensitivity, specificity, accuracy and precision, provides an excellent rationale for the incorporation of MS‐based assays into SES research investigations. However, the overall level and promptness for the integration of MS‐based analyses into SES have, perhaps, been lacking when considering the innate benefits it can add to the field. This is predominantly due to the specialist nature of the equipment, requiring extended technical training and dedicated research laboratories with the infrastructure to house large pieces of kit. This is coupled with the requirement for these labs to have facilities to generate pure gases (e.g. nitrogen) and appropriate access to building ventilation to remove exhaust gases from the operation of large (and noisy) rotary pumps necessary to maintain the equipment under a constant state of vacuum. Hence, MS is still predominantly performed in specialist laboratories, although smaller, more ‘focused’, MS laboratories are being frequently incorporated into bioscience research spaces. Despite these hurdles, it is evident from the research landscape that the use of MS is constantly evolving to spill out of the chemical sciences to continually increase its application across bioscience topics such as clinical chemistry,^2^ nutrition^3^ and systems biology^4^ amongst others.

MS instrumentation is available in many different formats, with the choice of the analyte(s) of interest and the field of the application being the main drivers for deciding the most appropriate MS approach to employ. For example, targeted analyses, that is where the researcher knows exactly what they want to measure, are often benefitted from the use of nominal resolution, tandem‐MS systems. Nominal resolution systems provide a measurement of the charged ion(s) which is generally accepted as the nearest mass unit integer (known as a Dalton or Da), with the greatest level of mass accuracy possible to identify the nearest 0.1 Da. However, this may not be specific enough to resolve compounds with similar *m/z* values. Tandem‐MS systems, often written as MS/MS, include the sequential use of multiple mass analyzers to add increased specificity through the measurement of precursor (i.e. intact molecules) and product (i.e. molecular fragments) ions. MS conditions can be optimized to allow a certain analyte to be chosen based on its precursor ion, fragmented through excitation in an inert gas setting, followed by the selection of a specific (and known) fragment ion(s) for final measurement at the instrument detector. This allows the removal of non‐relevant molecules which share a product ion *m/z* (or one close enough to be included on the filter) with the analyte(s) of interest. On the other hand, researchers who wish to follow an ‘exploratory’ (i.e. non‐targeted) analysis approach, where the analytes of interest are not known (i.e. hypothesis generating), need to be able to resolve between molecules with very similar *m/z* values for reliable analyses. For this reason, these experiments (which include approaches such as metabolomics, lipidomics and proteomics) employ the use of high mass accuracy and high‐resolution (commonly referred to jointly as high‐resolution) mass spectrometers capable of distinguishing ions with *m/z* values with as little as < 1 ppm (0.0001%) difference. This allows multiple compounds with similar *m/z* values to be differentiated from each other within the output data files, increasing the potential number of analytes capable of being measured in a single analytical run. Whilst it might sound appropriate to use the higher resolution instruments for all applications, in reality, the equipment is of extremely high comparative cost and (slightly) less amenable to high‐throughput, quantitative analyses when compared to the nominal resolution tandem‐MS systems. The addition of internal standards, often stable isotope‐labelled versions of the molecule of interest, aid to increase the reproducibility and robustness of assays for all instrumentation and methodological approaches. However, these labelled entities can be at a high cost and not all are available commercially and thus may need to be synthesized in‐house. In addition to the use of MS, many biological applications also include a form of chromatographic separation, with liquid chromatography (LC) and gas chromatography (GC) being the most commonly applied in human biomarker analyses. The inclusion of chromatography prior to MS analysis offers a way of reducing the complexity of analyzing sample mediums which contain a high number of different analytes. In SES, the major sample mediums analyzed include plasma, serum, and urine, all of which are extremely complex in nature. Chromatography allows for these complex mixtures to be separated out into less complex ‘bitesize’ pieces which means that the mass spectrometer is only required to process a smaller number of ions at any one point in time. This separation allows for the optimization of sensitivity and specificity and provides a time‐dependent trace of the analytes appearing in the analysis as a ‘peak’ which can be integrated to provide a semi‐ or fully quantitative result. This means that samples from different study groupings (e.g. exercise vs. non‐exercise) can be compared either through the concentration of identified analytes or via a comparative analysis of peak areas without specifically knowing the identification or concentration of the analyte.

This manuscript provides an overview of the applications of MS across studies investigating aspects related to sport or exercise included in manuscripts published, or currently in press, throughout 2022. The review intends to provide a comprehensive roundup of the work completed over the past year but is not intended to be fully exhaustive of all publications available across multiple indexing databases. In the interest of brevity, not all identified publications are discussed in detail but offer equal importance in their contribution to the field. Publications were identified through the PubMed and Google Scholar databases using the terms ‘mass spectrometry’ AND ‘sport OR exercise’ up until 31 October 2022, alongside articles identified through personal citation alerts sent to the manuscript authors. Any articles in which the full text could not be accessed and did not explicitly state the role of MS within the title/abstract were excluded, as were preprint submissions. Articles relating specifically to the use of MS in sports anti‐doping have been excluded as an excellent review of the most recent applications in the anti‐doping field can be found elsewhere.^5^ In addition, the articles included within this review pertain only to those including human research participants, with solely animal‐based studies excluded from the current discussion.

## 2 RECENT USE OF MS IN SES

Research studies in SES involve the use of an array of analytical techniques to provide biochemical information related to the sporting/exercise situation in which the participants are being studied. Examples of these analyses include cross‐sectional information (e.g. menstrual hormone status in female participants), interventional changes (e.g. pharmacokinetics of nutritional intake), biological responses (e.g. oxygen kinetics during incremental exercise), and associative outcomes (e.g. levels of muscle damage markers post‐exercise). MS, therefore, affords an excellent opportunity to expand the catalogue of biochemical markers available to the sports scientist, heavily expanding on the more traditional SES‐related biomarkers provided by plate‐based immunoassay approaches. This is not to say these approaches are not valid, nor redundant, but the opportunity to maximize the capacity for analytical science in SES is one that should be relished by applying a combination of techniques to provide the most comprehensive and, importantly, relevant directory of biomarkers. For example, the quantitative measurement of larger protein structures, such as cytokines and growth factors, by MS requires complex analytical approaches to ensure reliable analyses both for intact and peptide‐digested protocols. These methods require the more expensive end of instrumentation, along with highly trained personnel and costly reagents (e.g. isotopically labelled peptides). For this reason, it would be advised to use commercially available and validated plate‐based assays. Nonetheless, if a study requires the specific identification of parent and fragment peptide chains, e.g. distinguishing neuropeptide Y 1‐36 and 3‐36 in the study by Eugster et al.^6^ included in this review, then an approach using MS is required to provide a sufficient level of assay specificity.

Exercise interventions are not only linked to sports or sporting activities. In the modern day, there is a concerted push to increase the amount of physical activity that individuals are completing to act as a protective mechanism for a range of diseases, notably those related to metabolic disturbances such as type II diabetes mellitus (T2DM). This has been termed as ‘Exercise as Medicine’,^7^ with weekly guidelines for the amount of physical activity being set by organizations such as the American College of Sports Medicine (ACSM).^8^ The information provided by analysis of clinically relevant biomarkers can provide important insight into the general health of the individual, as well as the direct impact of exercise interventions on a clinical condition. As an example, a randomized control trial involving colorectal cancer patients utilized MS to quantitate urinary 8‐oxo‐2′‐deoxyguanosine, a marker of oxidative deoxyribonucleic acid (DNA) damage and showed that a short‐term moderate‐intensity (but not high‐intensity) exercise program was able to reduce levels in patients.^9^ These data, coupled with a physical investigation, can be a powerful tool to understand the health benefits of exercise and inform on the (patho)physiological activities of the participant in relation to the dynamic profiles of metabolites and/or proteins present in biosamples. Altogether, this represents an important contribution that MS can offer to exercise‐based research to facilitate the understanding and improvement of global health and disease, with a view to accelerating the protective and therapeutic understanding of physical activity and the underpinning physiological and biochemical mechanisms.

**FIGURE 2:**
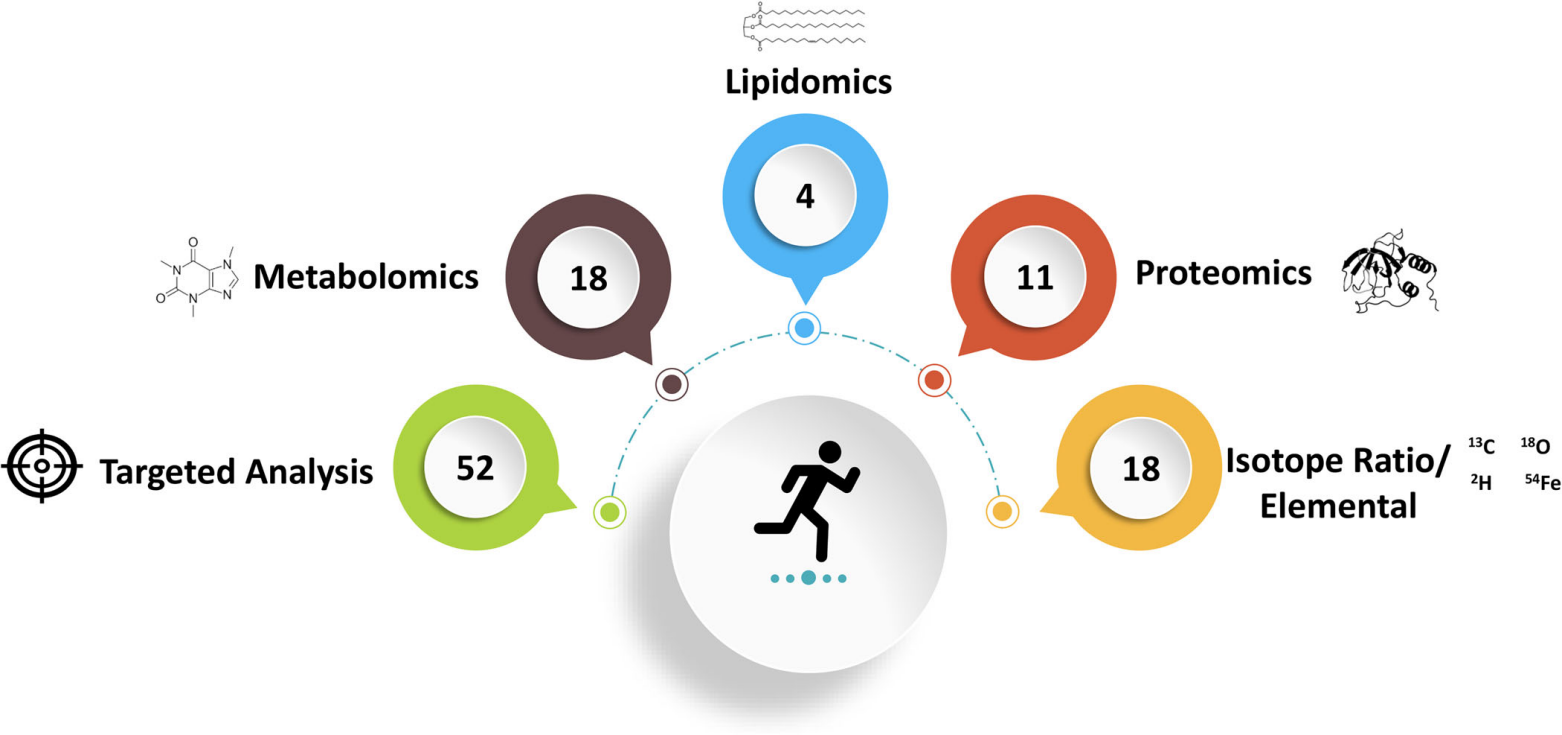
Number of articles included in this review relating to the use of mass spectrometry in sport and exercise science split by analytical approach. Note that some publications applied more than one approach and validation studies are not included within the values; therefore, the total number does not equal the 93 publications stated to be included in this review.

In total, we identified 93 publications in 2022 related to the use of MS in SES which are included in this annual review. Articles identified via database searching or citation alerts were initially screened by title and abstract, followed by a brief investigation of the methods sections to ensure that both the use of MS and a focus on SES were present. Articles that met these criteria were read in entirety and classified according to their analytical approach and split into targeted analyses, metabolomics, lipidomics, proteomics, and other studies which applied isotope ratio (IR) and elemental MS approaches. A brief explanation of what is meant by these different analytical approaches is included within each respective sub‐section. The number of studies identified in each of these categories can be seen in Figure 2, with more information for each study provided in the Supporting Information. In addition, six publications collected biosamples from sporting populations solely for use in the optimization and/or validation of an MS‐based assay; these publications will not be discussed in this review but are included for reference.^10^, ^11^, ^12^, ^13^, ^14^, ^15^ These studies represent work across a broad range of participants and environments, including but not limited to work completed in athletes, military personnel, medical/hospital patients, and comparisons across young and old persons. Overall, it is exciting to see the gradual increase in the application of MS in SES, with these papers herein highlighting the progress made over the previous calendar year.

## 3 ANALYTICAL APPROACHES FOR MS‐BASED INVESTIGATIONS IN SES IN 2022

### 3.1 Targeted analysis

Targeted analyses allow the researcher to collect information on a specific molecule or set of molecules related to the research hypothesis. Examples of where this approach is useful include the quantitative change of certain biochemicals in response to an intervention, a cross‐sectional analysis of biomarker levels in a relevant population, and to test the bioavailability of a nutritional supplement, that is, does the supplement enter the systemic circulation, and for how long does it remain in its unmetabolized form. In 2022, targeted analyses were the most commonly applied approach for MS‐based research in SES, with 52 human participant studies published. These experiments were predominantly performed in plasma^6^, ^16^, ^17^, ^18^, ^19^, ^20^, ^21^, ^22^, ^23^, ^24^, ^25^, ^26^, ^27^, ^28^, ^29^, ^30^, ^31^, ^32^, ^33^, ^34^, ^35^ or serum,^36^, ^37^, ^38^, ^39^, ^40^, ^41^, ^42^, ^43^, ^44^, ^45^, ^46^, ^47^, ^48^, ^49^, ^50^ with other biosamples mediums utilized such as urine,^51^, ^52^, ^53^, ^54^, ^55^ fecal matter,^56^, ^57^ breath gases,^58^, ^59^, ^60^, ^61^, ^62^, ^63^ red blood cells,^64^ cerebrospinal fluid,^25^ hair,^65^ and whole blood via microsampling.^66^ This excludes the use of IR and elemental MS to target specific isotope/elemental signatures, which are discussed individually within section 3.5.

#### 3.1.1 Vitamin D

Vitamin D metabolites were a commonly investigated target in 2022, being investigated in six separate publications.^22^, ^31^, ^37^, ^43^, ^48^, ^49^ Three of these studies were cross‐sectional in design,^22^, ^31^, ^37^ with the remaining studies applying dietary and/or exercise interventions to target vitamin D status.^43^, ^48^, ^49^ Vitamin D is a group of prohormones that play an important role in maintaining serum mineral status (notably calcium (Ca^2+^) and phosphate (PO_4_^−^) ions) which is integral to the formation and maintenance of bone mineral density.^67^ There is also a consideration for vitamin D to improve aspects of exercise function/performance, with an indication in older populations showing a strong positive association,^68^ albeit the evidence to support this within an athletic population has been mixed and therefore there is currently no consensus on its efficacy in sport.^69^ Precursor vitamin D molecules are biologically inactive, namely vitamin D_2_ (cholecalciferol) and D_3_ (ergocalciferol) and must be converted downstream to active metabolites calcidiol [25(OH)D] and calcitriol [1,25(OH)_2_D], prior to conversion for excretion as 24,25‐dihydroxycholecalciferol [24,25(OH)_2_D].^67^ Calcitriol has been previously identified to show an apparent role in activating muscle synthesis pathways,^70^ and therefore provides an appropriate rationale for the potential to improve muscular and/or endurance performance. To this end, a cross‐sectional study from this year reported that MS‐based measurements of serum vitamin D metabolites were associated with 2.4 km run time [25(OH)D, 1,25(OH)_2_D, and 24,25(OH)_2_D], as well as muscular strength [1,25(OH)_2_D] and power [25(OH)D and 1,25(OH)_2_D].^37^ Interestingly, improved run times, along with increased muscular strength and power, were associated with a proportionally greater conversion of active vitamin D metabolites to the inactive excreted form, 24,25(OH)_2_D, indicating a potential for the inter‐individual processing of vitamin D metabolites to be associated with measures of physical performance. In a study performed on children (6–8 years), researchers assessed the combined (and separate) effects of supplementing with vitamin D and protein during the winter period on various measures of muscular strength and physical performance.^49^ Vitamin D supplementation had no overall effects on parameters of strength or function, although greater improvements in leg strength with vitamin D supplementation were observed in the girls compared to boys. The reasons for this are unclear but may indicate potential sex differences in response to vitamin D supplementation that requires future investigation. In another study in children, a longer time spent sedentary coupled with lower consumption of fortified dairy products was linked to vitamin D deficiency, indicating the potential importance of physical activity/exercise in maintaining nutrient status alongside dietary intake.^31^ Lastly, vitamin D levels during pregnancy were investigated alongside a combined walking exercise (10,000 steps/day and 4 × 40 m walks/week) and a high dairy dietary protein intake protocol.^48^ The intervention led to lower bone resorption and increased bone formation markers, although the levels of vitamin D metabolites were not different between groups at any time point. Although results in vitamin D status and physical activity/exercise have continued to show contrasting results, the importance of measuring vitamin D metabolites remains. Historically, 25(OH)D [a combination of 25(OH)D_2_ and 25(OH)D_3_] was measured by radioimmunoassay or competitive protein binding assays. However, these assays have indicated many technical issues with reproducibility across sites being poor,^71^ with the consensus to switch vitamin D assessments to LC‐MS methods to provide specific and sensitive quantitation of individual metabolites.^72^ This demonstrates a vital role for MS to continue its involvement within this research topic area.

#### 3.1.2 Gut microbiome

An area of research which has gained accelerating interest within the exercise sciences is the influence of the gut microbiome and the metabolites produced by gut bacteria on health and exercise performance. One of these metabolites is urolithin A, a polyphenolic breakdown product of ellagic acid and other ellagitannins which are present in foods such as pomegranates, berries, teas, and nuts.^73^ This metabolite, found in multiple biofluids and tissues, has been observed to increase mitophagy and mitochondrial function whilst also reducing inflammation, demonstrating positive protective effects in pre‐clinical studies of age‐related conditions.^74^ This has garnered an interest in the potential for urolithin A to improve age‐related declines in muscular function. Two studies in 2022,^18^, ^24^ led by the team at Amazentis in Switzerland, investigated the impact of a four months supplementation with urolithin A (Mitopure, Amazentis, Switzerland) and demonstrated increases in muscular strength, peak oxygen uptake (known as VO_2peak_), and 6‐min walking test scores, but not peak power output, in middle‐aged men (40–64 years).^18^ A similar study performed in older men (60–90 years) was not able to replicate the positive impact on results for 6‐min walking test scores, nor demonstrated any improvement in adenosine triphosphate (ATP) production in hand skeletal muscle.^24^ However, supplementation with urolithin A for two months was associated with improved muscular endurance in the tibialis anterior and first dorsal interosseus, although these improvements against the placebo trial were diminished by month four. These studies utilized a targeted LC‐MS method to measure circulating urolithin A and its glucuronide metabolite to show dose‐dependent increases in basal levels across the supplementation period. In a similar consideration to urolithin A, there is a developing interest in short‐chain fatty acids (SCFAs) within the SES field. SCFAs are bacterial metabolic by‐products of non‐digestible starches and proteins, with acetic acid (acetate), propionic acid (propionate), and butyric acid (butyrate) the most abundantly produced within the gut.^75^ These molecules have been identified to act as prominent anti‐inflammatory and anti‐fibrotic mediators in pre‐clinical cellular/animal models,^76^ and are postulated to provide potential benefits to exercise performance via these anti‐inflammatory and potentially pro‐immune mechanisms.^77^ Three studies included in this review investigated SCFA levels in plasma^16^ and faeces.^56^, ^57^ Williams et al.^56^ examined the impact of oligofructose‐enriched inulin intake on the gut microbiome and VO_2peak_ response to high‐intensity interval training. The authors noted no beneficial effects on exercise performance, with an indication that the intervention increased total SCFA production and improved gut microbial levels of beneficial bacteria, albeit these results were deemed as not statistically different following adjustments for false discovery rate. Secondly, Torquati et al.^57^ assessed the impact of combined (aerobic and resistance) moderate‐intensity interval training versus a high‐intensity protocol in T2DM patients. Although the exercise intensity influenced the gut microbial abundance and metabolic function, there were no differences in SCFA production across the groups. Finally, a study in healthy, active women explored the response of the circulating endocannabinoidome (eCBome) mediators and SCFAs to acute maximal aerobic exercise after seven days of eating a Mediterranean‐style diet rich in n‐3 fatty acids or a control diet.^16^ The use of LC‐MS/MS allowed for the quantitation of a broad spectrum of endogenous bioactive lipids, some of which were shown to be significantly upregulated for up to 1‐h post‐maximal aerobic exercise, including increases in *N*‐acylethanolamines and polyunsaturated fatty acids in the Mediterranean‐style diet trial, with little to no changes in the fecal microbiome or SCFA levels. This study demonstrated that the eCBome responds to acute aerobic exercise in a manner specific to the diet within the preceding week, potentially highlighting an influence of eCBome signalling in exercise‐induced metabolic responses to exercise in females.

Overall, The study of the microbiome and its metabolic by‐products in relation to human physiology is currently a hot topic across multiple research fields. This has been driven by investigations in clinical medicine^1^ but has shown promising applications to SES. The investigation of circulating small molecule metabolite levels produced by the gut bacteria, such as urolithin A and SCFAs, is innately benefited from the availability of MS‐based assays; therefore, the capacity for SES researchers to access MS instrumentation to measure these molecules will be of direct benefit to the field.

#### 3.1.3 Post‐exercise recovery

Rapid recovery from intense exercise is paramount for optimal exercise performance, therefore, investigation of the vagal reactivation pathway is an important area of research within SES. Neuropeptide Y (NPY) is a 36‐amino acid peptide released in response to physical stress and is involved as a co‐factor of catecholamine secretion and blood pressure control. Eugster et al.^6^ conducted a study in this area to elucidate whether NPY presence can activate the vagal pathway following exercise cessation to reduce catecholamine secretion from the sympathetic neurons in the peripheral autonomic system. The study included both high‐intensity (120% ventilatory threshold‐1; VT1) and moderate‐intensity (80% VT1) exercise with NPY fragments (NPY1‐36 and NPY3‐36) analyzed alongside physical (e.g. breath gas kinetics) and biochemical (e.g. norepinephrine) measurements. NPY fragments were independently quantitated using a micro‐LC‐MS/MS approach. The authors identified that the level of NPY was dependent on the intensity of exercise and that NPY 1‐36 had a significantly shorter half‐life in vivo than previously considered, with the majority of the biochemical activity being driven by NPY 3‐36. This offered a finding made possible only through the use of MS. From an exercise perspective, NPY3‐36 did not potentiate recovery, although it was considered to potentially inhibit acetylcholine secretion, which subsequently affects vagal bradycardia. Additionally, an inhibiting dipeptidyl peptidase 4 blockade (to inhibit NPY1‐36 breakdown to NPY3‐36) caused no alteration to the vagal reactivation during the post‐exercise recovery period, thus the regulatory mechanisms of NPY and its specific peptide metabolites remain unclear, especially as reduced NPY 3‐36 levels did not correspond to increased NPY 1‐36.

#### 3.1.4 Physiological consequences of exercise training

**FIGURE 3:**
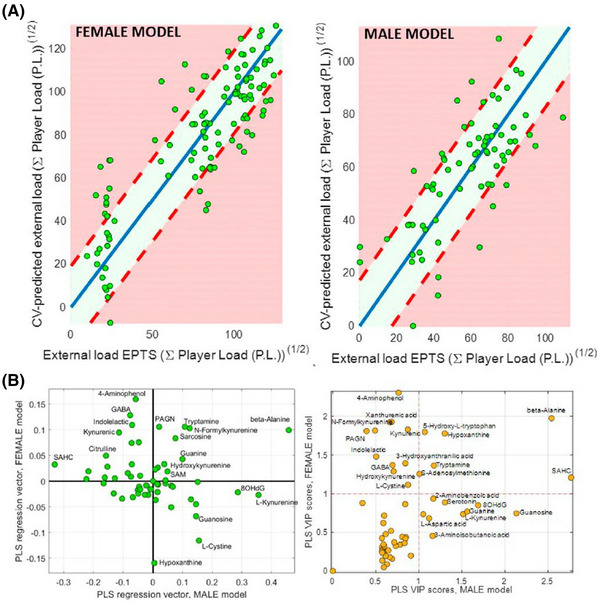
(A) Cross‐validation of external load predictions calculated by partial least squares (PLS) analysis of urinary metabolic compounds in professional soccer players. (B) PLS regression vectors (left) and variable importance in projection (VIP) scores (right) demonstrating a lack of correlation between female and male players. Reproduced with permission from Rodas et al.^52^ (Copyright 2022 Rodas, Ferrer, Reche, Sanjuan‐Herráez, McCall and Quintás, CC‐BY). https://creativecommons.org/licenses/by/4.0/.

In sports research, there has been an increasing interest in the potential for MS to identify biological compounds that can be used as surrogate markers to indicate training adaptations. To this end, Rodas et al.^52^ followed professional soccer players throughout a season, collecting urine samples from female and male players across different playing positions. Samples were collected at various times across the season and at the beginning of the pre‐season in the female cohort. A large‐scale LC‐MS‐based targeted assay approach was used to measure a range of amino acids and metabolites related to tryptophan and phenylalanine pathways. Two metabolites demonstrated linear relationships with cumulative external load in both the female and male teams. β‐alanine showed a positive association with cumulative external load in both teams, whereas s‐adenosylhomocysteine showed a positive association in males and a negative association in females. When investigating the cumulative training load using a multivariate approach (i.e. building a prediction model by including all measured metabolites), the authors noted the capacity to predict external load values in both the female and male players by using the metabolic data. This supported the hypothesis that the urinary metabolome would demonstrate quantitative shifts across a competitive season, indicating a potential for metabolite values to associate with physiological adaptation, although the current study was not able to effectively demonstrate this conclusion. Interestingly, the multivariate models developed to predict external load showed distinct cross‐sex differences, indicating that metabolic shifts within competition are not appropriate as a one‐size‐fits‐all approach and must be tailored depending on sex (Figure 3). Although this study only looked at soccer players from one professional club in Spain, it may be that future research demonstrates different metabolic shifts dependent on factors such as athlete location, diet, and discipline, amongst others.

In another study by Kosaki and colleagues,^29^ concentrations of plasma hypoxanthine, xanthine, and uric acid were shown to be increased following the cessation of a marathon in association with elevated plasma xanthine oxidoreductase activity. Interestingly, the magnitude of elevation associated with these markers was increased in individuals who also demonstrated indices of acute kidney injury (AKI), including increased serum creatinine and urinary liver‐type fatty acid binding protein levels. The potential mechanisms involved in transient exercise‐induced AKI have yet to be fully understood; however, measurements of xanthine oxidoreductase and associated metabolites may provide insights into the pathogenesis of renal impairments after marathon running.

### 3.2 Metabolomics

Metabolomics involves the study of small molecule metabolites which are processed/secreted by a living organism.^78^ Altogether these metabolites make up the ‘metabolome’, a term used to describe the full array of metabolites present within the system.^79^ The process of metabolomic analysis predominantly involves the use of chromatography coupled to high‐resolution MS systems to probe the metabolomic signatures of experimental biosamples. This allows the characterization of a large number of peaks (or features) that may represent specific metabolites within the system. This approach has been widely applied in SES over the past decade, with a major focus on identifying cross‐sectional information on athlete populations,^80^ as well as pre‐ and post‐exercise changes in metabolic profiles.^81^ Metabolomics follows a non‐targeted methodology where the ultimate goal is to provide a comprehensive identification of all possible metabolite changes, with an attempt to introduce as little bias as is feasible. This allows the researchers to investigate the data for information relating to qualitative (i.e. presence/absence) and/or semi‐quantitative (i.e. a peak area is produced with no formal concentration calculation) outcomes. Naturally, there always remains some level of bias related to the sample type, preparation steps, column chemistry (i.e. polar vs. non‐polar retention), and MS conditions (e.g. positive or negative ionization) where the overcoming of these aspects may only be achieved through multiple iterations of sample analyses. Despite metabolomics generally referring to the investigation of the metabolome in a non‐targeted manner, there has been a concurrent increase in the widescale targeting of metabolites to provide information on a large number of known targets, albeit the assay is without specific quantitative validation for all/any of the compounds of interest.^82^ These methods generally find themselves to include the metabolomic terminology but are perhaps better considered only as targeted investigations as there is a biased perspective to what is being measured. As an example from this past year, Puigarnau et al.^66^ discussed the use of metabolomics to show varied changes in metabolites related to an amino acid, purine, and nitrogen metabolism. However this study, in fact, used an entirely targeted approach where the authors described the optimization of each metabolite using chemical standards followed by a tandem‐MS assay; although, the authors showed no data to demonstrate the assay was valid for targeted use (i.e. lack of data for accuracy, precision, matrix effect, etc.). Nonetheless, the authors claim the application of a multi‐metabolite profiling panel to be of use for investigating training load in runners. This wideband targeted approach was also identified in six additional studies over the past year.^25^, ^26^, ^28^, ^40^, ^52^, ^53^

**FIGURE 4:**
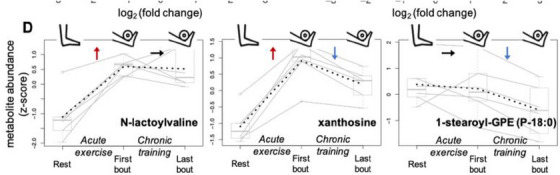
Measured levels of three selected metabolites across a 4‐week resistance exercise program showing changes following acute (*N*‐lactoylvaline), chronic (1‐stearoyl‐GPE (P‐18:0), and acute/chronic (xanthosine) training. Adapted with permission from Gehlert et al.^85^ (Copyright 2022 by the authors, CC‐BY). https://creativecommons.org/licenses/by/4.0/.

In 2022, 18 studies used a traditional non‐targeted metabolomics assay.^18^, ^24^, ^30^, ^83^, ^84^, ^85^, ^86^, ^87^, ^88^, ^89^, ^90^, ^91^, ^92^, ^93^, ^94^, ^95^, ^96^, ^97^ Perhaps the most thought‐provoking exercise metabolomics publication from 2022 came from the Long Lab at Stanford University and was published in *Nature*. This study identified an exercise‐induced metabolite, namely *N*‐lactoylphenylalanine (Lac‐Phe), to be associated with reduced dietary intake.^30^ The authors began their investigations by assessing the plasma metabolome of mice and horses to compare analytical features pre‐ and post‐exercise. In both animal species, they identified the same feature (*m/z* 236.0928, [M‐H]^−^) to be highly associated with an increase in post‐exercise samples. The feature was then identified as Lac‐Phe through the analysis of a synthetic Lac‐Phe standard which demonstrated a match in *m/z*, retention time, and fragmentation profile. This provides an excellent example of where an unknown feature has been formally identified and a quantitative assay is then developed for further targeted analyses. Further to the initial finding of Lac‐Phe increasing with exercise, the research team demonstrated that the administration of Lac‐Phe to diet‐induced obesity mice decreased food intake leading to a reduction in body weight, glucose response to an oral tolerance test, and intra and extracellular white adipose tissue, all despite no differences in overall activity levels between the mice administered Lac‐Phe or vehicle control. Although this study was originally conducted in animal models, the authors later went on to confirm a similar change in post‐exercise Lac‐Phe levels in humans. They observed that levels were risen in endurance, resistance, and sprint exercise, with the latter showing the most pronounced increases during and for up to 1‐h post‐exercise and heavily associated with concurrent increases in plasma lactate levels. Similarly to this, Gehlert et al.^85^ investigated the metabolome of the vastus lateralis (a muscle within the quadriceps group) at three‐time points across a four‐week resistance exercise training program (prior to, after the first session and after the last session). Resistance exercise training altered 33 metabolites in total, with increases in 3‐methylhistidine, a biomarker for skeletal muscle breakdown. Interestingly, one of the metabolites shown to increase with resistance exercise activity was *N*‐lactoylvaline (Lac‐Val) (Figure 4), another lactate‐amino acid derivative similar to Lac‐Phe identified in the previously mentioned study. This identifies lactate‐derived di‐peptides as an interesting set of targets for future exercise‐induced metabolic investigations.

#### 3.2.1 Steroidal profiling

**FIGURE 5:**
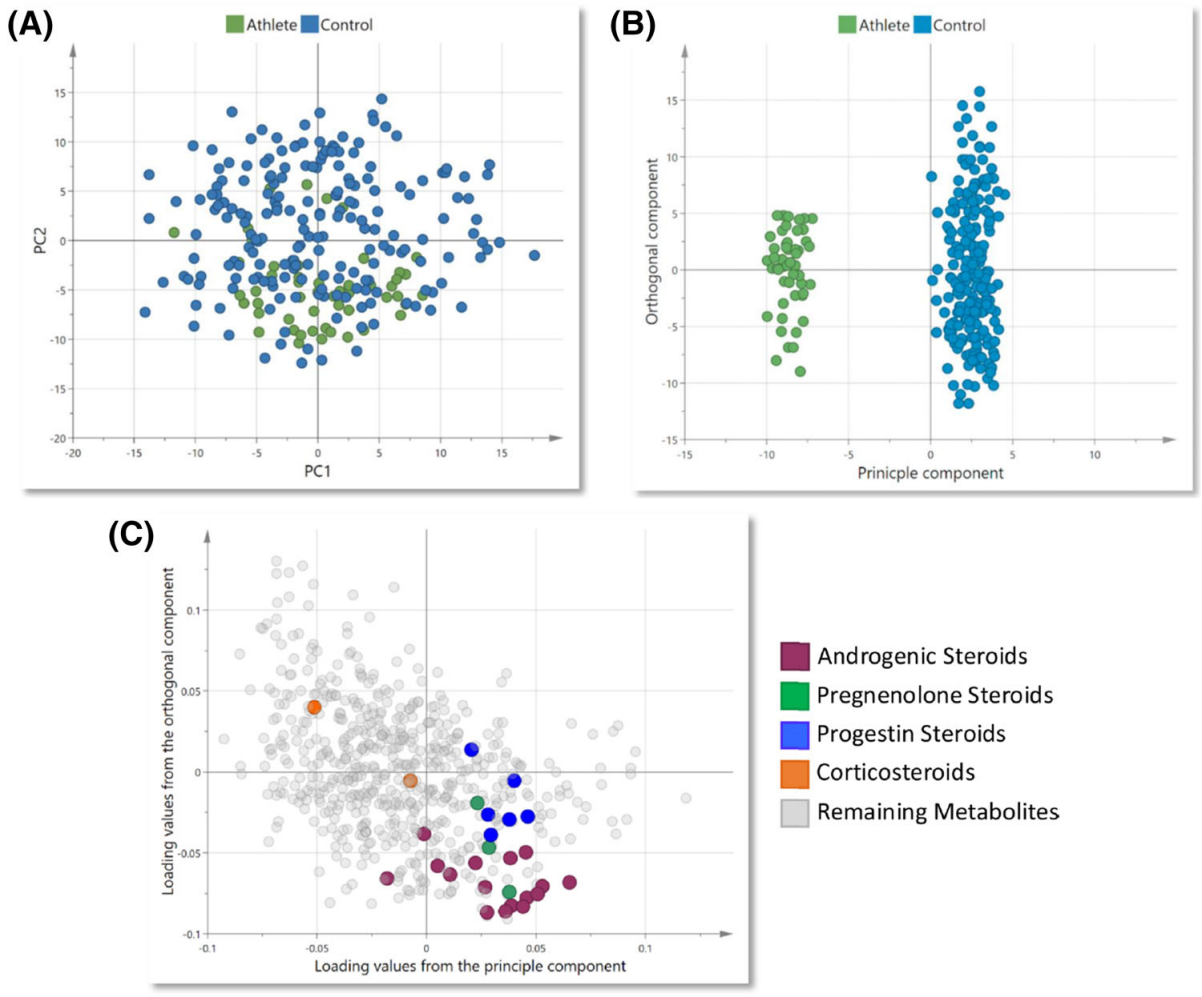
(A) Principal component analysis (PCA) of metabolite levels in elite female endurance athletes and non‐athletic controls. (B) Orthogonal partial least squares discriminant analysis (OPLS‐DA) identifying differences in overall metabolic profiles between elite female endurance athletes and non‐athletic controls. (C) Loadings plot corresponding to the OPLS‐DA model showing the influence of steroid hormones classes (notably androgenic steroids) on differences in profiles between elite female athletes and non‐athletic controls. Adapted with permission from Tarkhan et al.^97^ (Copyright 2022 The Authors, CC‐BY). https://creativecommons.org/licenses/by/4.0/.

Steroidal profiling via MS measurement offers an important surveillance technique in the sports anti‐doping sector. To this end, in 2014 the steroidal module was added to the World Anti‐Doping Agency's (WADA) Athlete Biological Passport to facilitate a longitudinal assessment of six steroid hormones/metabolite derivatives.^98^ However, there is a need to better understand the discriminating metabolic changes in steroid hormones that are unrelated to doping, as well as gain a better understanding of steroid profiles and dynamics in transgender athletes and those with differences in sexual development. One study that recently investigated this area used an MS‐based metabolomic approach to investigate metabolic differences between female elite endurance athletes and non‐athletic control participants.^97^ From this non‐targeted approach, the authors identified that elite athletes had reduced levels of androgenic, pregnenolone, and progestin hormones, with elevated corticosteroid levels when compared to non‐athletic individuals (Figure 5). The utilization of this non‐targeted approach allowed the assessment of samples from multiple different sports (athletics, boxing, rowing, cycling, kayaking, swimming, hockey and tennis), to confirm that elite female endurance athletes have a distinct steroid hormone profile which may be relevant to doping control investigations. Aside from the doping angle, female athletes are at high risk of developing relative energy deficiency in sport^99^ with or without disordered eating, which may increase the risk of hormone deficiency^100^ which was identified in this study as a potential scenario due to lower steroid hormone levels in the elite population.^97^

#### 3.2.2 Metabolic profiling and health

Over the past year, exercise/sport‐based metabolomics experiments have further improved our knowledge of the relationship between endogenous metabolic profiles and health. For example, Tso and colleagues^83^ collected plasma samples from freshman collegiate American football players during pre‐season training and 5 months later to coincide with the cessation of the competitive season. The investigators wished to assess metabolite profiles against adaptations/changes in cardiovascular phenotype across the period. This included the measurement of left ventricular mass index and diastolic function. They identified that improved cardiovascular parameters were associated with increases in arginine and a decrease in hypoxanthine and saturated fatty acids, with increases in lysine and pipecolate indicating adverse cardiovascular health. Similarly, Zhao et al.^89^ investigated metabolic health but in a non‐sporting population, observing metabolomic changes following an exercise intervention in individuals diagnosed with chronic fatigue syndrome. The exercise intervention was designed to align with the aforementioned ACSM guidelines.^8^ Morning urine samples were analyzed by GC‐MS prior to and following the intervention and showed that exercise training was able to decrease symptoms of fatigue, associated with changes in putrescine, 6‐phospho‐D‐gluconate and pentose levels.

#### 3.2.3 Reporting standards

Although much of the previous exercise/sports‐focused metabolomics experiments have been hypothesis‐generating in nature, the constant improvement in analytical and data processing methods means that many of the previously unidentifiable metabolomic features are now able to be confidently assigned as specific compounds. Nonetheless, there is still a lack of reporting standards within this community on exactly how, and to what extent, the identification of the metabolites has been completed. This problem is compounded by the use of external, commercial entities who provide analysis and identification, but will not provide any raw data or specific information on how the analytes were identified. For this reason, the SES field must better address the reporting standards for metabolomic data analysis. It is imperative for manuscript editors and reviewers to request evidence as detailed by the Chemical Analysis Working Group as part of the Metabolomics Standards Initiative.^101^ This initiative builds upon the reporting for the aspects of metabolomics experiments, including sample preparation, experimental analysis, quality control, metabolite identification, and data pre‐processing. This will provide editors, reviewers, and readers of manuscripts with the experimental overview and completeness of data to improve the quality of information released into the public domain.

### 3.3 Lipidomics

Lipidomics analysis falls under a sub‐section of metabolomics where lipid species are specifically targeted. Lipids are essential molecules for a variety of biological functions including, but not limited to, energy production and storage, cellular signalling, vitamin absorption, and hormone production. These aspects are all important factors in SES whether focusing on exercise interventions for health and disease or for the optimization of fat utilization in performance sports. Historically, an interest in lipids (also referred to as fats) has been based on overall levels/groups of these species and their associations with health and exercise characteristics. The applied methodological approaches of modern‐age lipidomics help differentiate between lipid classes and, in some cases, are specific enough to identify lipid chain structures including double bonding and side chain locations which may be important to classify the source of the lipid or the biological process in which it was synthesized. This allows researchers to study the flux of complex biological systems more appropriately to further understand the metabolic impact of exercise across varied populations.^102^ Lipidomics remains an emerging method within SES research but, nonetheless, is providing data to improve the general understanding of lipid metabolism in exercise‐based scenarios.

**FIGURE 6:**
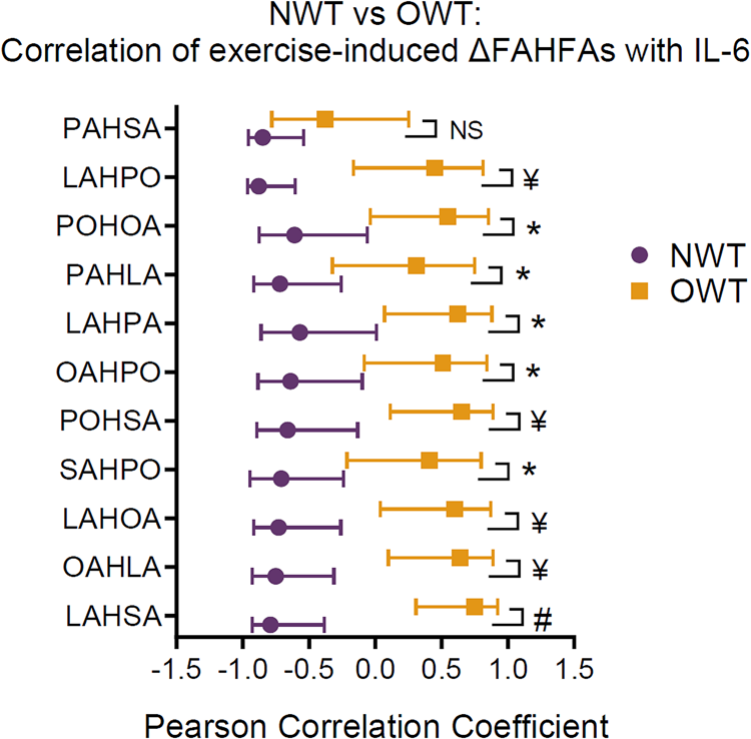
Pearson correlation coefficients of exercise‐induced changes in fatty acid esters of hydroxy fatty acids (FAHFAs) against interleukin‐6 (IL‐6). Symbols indicate a significant difference (adj. *p* < 0.05) in Pearson's rho between normal weight trained (NWT) and overweight trained (OWT) groups. Adapted with permission from Nelson et al.^87^ (Copyright 2022 Nelson et al., CC‐BY). https://creativecommons.org/licenses/by/4.0/.

In 2022, four studies were published using an exercise/sport‐based lipidomic approach, with two publications investigating obese subjects following aerobic and resistance exercise protocols,^103^, ^104^ one comparing trained individuals who were either overweight or normal weight,^87^ and one involving human immunodeficiency virus‐positive patients.^105^ San Martin et al.^104^ prescribed combined physical training (aerobic and resistance) to obese females in a protocol including three sessions of alternating modalities each week for eight weeks. Non‐targeted lipidomics analyses identified increases in three lysophosphatidylcholines and arachidonic acid, with decreases in three phosphatidylcholines. These changes in lipids were associated with increased physical fitness and reduced waist circumference. The authors concluded that these lipids might be involved in molecular mechanisms of obesity and exercise, but information from this individual study would not be able to draw these conclusions without further investigation. Similarly, the non‐targeted lipidomic analysis showed obese patients with post‐intragastric balloons benefitted from maintenance of body weight by performing moderate aerobic and resistance exercise, showing reductions in free‐fatty acids (mainly saturated, mono, and omega‐6 fatty acids).^103^ This study utilized the Sciex SelexION differential ion mobility separation device, also commonly known as high‐field asymmetric ion mobility spectrometry, or FAIMS. This technique is performed at atmospheric pressure, in the gas phase, and provides separation of analytes based on their mobilities in high and low electric fields.^106^ This allows an additional, or independent, separation of analytes being introduced into the mass spectrometer which is not based on chemical affinity such as in chromatography, thus providing an extra level of selectivity and the capacity to greatly increase sensitivity through the reduction of chemical noise. Aside from the studies in non‐athletic populations, Nelson and colleagues^87^ demonstrated in trained individuals that baseline levels of fatty acid esters of hydroxy fatty acids (FAHFAs) and free fatty acids were diminished in the overweight group compared to normal weight participants. Furthermore, samples collected following a 90‐min treadmill run showed that the normal weight group had a four‐fold decrease in FAHFAs which negatively correlated with circulating interleukin‐6, an observation which was not reported in the overweight group suggesting that FAHFAs could modulate the inflammatory response and fuel utilization (Figure 6).

Although lipidomics is popular in the modern scientific era, the expanse of its use in exercise‐based research is still some way behind that of the more established approach of metabolomics. Nevertheless, lipidomics has now proved itself as a regular technique in clinical research^107^ and, therefore, it is expected that sports/exercise‐based research investigations will continue to grow over the coming years. One area where the translation of lipidomics experiments to SES research can be facilitated is through the transition from non‐targeted to targeted assays. This would necessitate the inclusion of a wide range of internal standards to allow the absolute quantitation of lipid species, providing a more effective approach to allow cross‐study/cohort comparisons in lipid metabolite concentrations. However, it is important to reiterate that despite the approach used, scientists performing lipidomics experiments must effectively report their protocols and data. This has led to a recent publication from a panel of lipidomics scientists describing the lipidomics minimum reporting checklist.^108^

### 3.4 Proteomics

Proteins are an essential aspect of routine biological function, with cellular processes utilizing proteins for an array of actions such as initiating/regulating growth and repair, transporting and storing molecules, regulating energy production, cellular signalling, immune regulation, biofluid buffering and oncotic pressure maintenance, to name but a few. Naturally, this provides an important stimulus for SES research as these protein functions are imperative to performance (e.g. energy production, muscle growth), recovery (e.g. inflammatory responses), and immune surveillance (i.e. to fight off infections). It is, therefore, unsurprising that MS‐based proteomics has taken a driving role in SES research over the past two decades.^109^ Much like the previously discussed *‘omics’* methods, proteomics provides a non‐targeted profile of proteins present within a sample. These analyses are usually performed following a ‘bottom‐up’ approach; this refers to the analysis of proteins by measurement of their surrogate peptide fragments following proteolysis, often termed ‘shotgun proteomics’.^110^ Once analyzed, specialist software packages and databases allow the putative identification of proteins present in the sample based on the number of peptide sequences that are detected and present within a specific protein. The confidence of protein presence can be increased by identifying multiple peptide sequences that are unique to the proteolytic signature of that specific protein, providing the researchers with a protein identification to assign to their data. The peak areas generated by the LC‐MS(/MS) analysis of the peptide sequences can then be combined to provide a semi‐quantitative value to aid in the investigation of transient changes or ratios of proteins in a sample set. Similarly to targeted and metabolomics/lipidomics methodologies, guidelines for the reporting of proteomics information and data have been proposed by a group of experts as part of the minimum information about a proteomics experiment initiative.^111^

**FIGURE 7:**
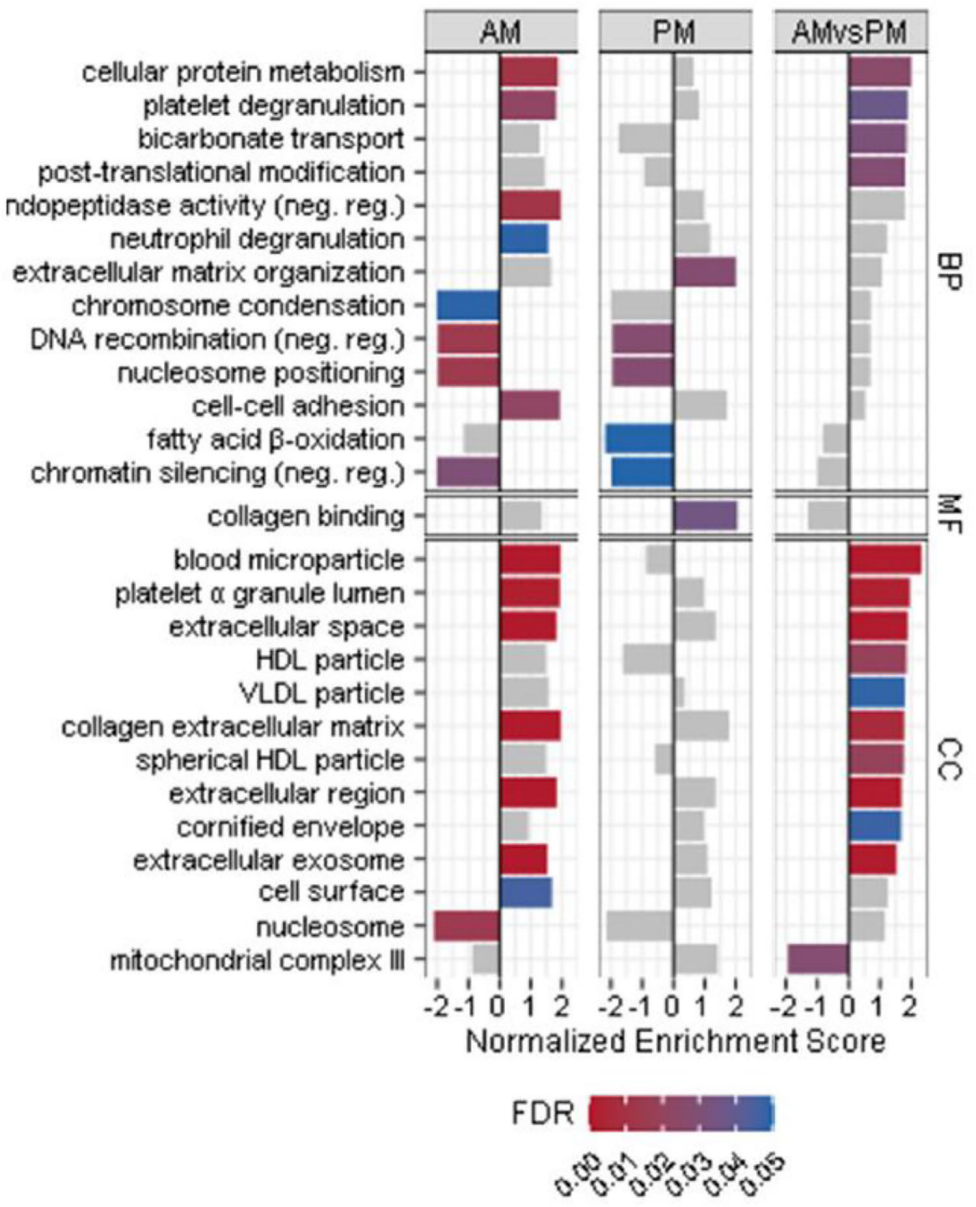
Normalized values for changes in skeletal muscle protein gene ontology set enrichment to assess log2 fold changes following morning (AM) or early evening (PM) training interventions. Bar colours refer to the false discovery rate (FDR) values. Adapted with permission from Savikj et al.^96^ (Copyright 2022 The Authors, CC‐BY). https://creativecommons.org/licenses/by/4.0/.

In 2022, proteomics was performed in 11 studies in the SES field, demonstrating a reduced application to metabolomics which is perhaps due to transient changes in method popularity. These studies were conducted using muscle tissue,^18^, ^96^, ^112^, ^113^, ^114^, ^115^ plasma,^116^, ^117^ saliva,^118^, ^119^ and urine.^120^ Vanderboom et al.^114^ performed a multi‐omics experiment to investigate the proteome, phosphoproteome, and transcriptome of the vastus lateralis muscle in lean and obese individuals who had performed 30‐min of single‐leg cycling at 70% VO_2peak_. The authors revealed that the obese participants displayed proteomic signatures which reflected reduced levels of mitochondrial function, and protein and glycogen synthesis, with markedly different exercise‐induced proteomic responses including notable identification of casein kinase II subunit alpha and glycogen synthase kinase‐3 beta pathways altered by obesity. They concluded that further investigation of the muscle proteome (and transcriptome) could lead to a better understanding of the mechanisms that could limit exercise‐induced pathway responses in obese individuals. Another study similarly assessed exercise‐induced proteome changes, albeit this time in the saliva of young adults completing a 40‐min walk/rest protocol at 5‐min intervals.^119^ One of the major aims of this study was to assess the differences in salivary protein content with stimulated (following chewing) or non‐stimulated collection modalities. However, additional data generated from the exercise intervention demonstrated that the low‐intensity intermittent walking protocol was able to upregulate salivary proteins related to immunity. These included various isoforms of immunoglobulins, lysozyme C, mucin‐7, and cystatin‐S, amongst others. A study by Savikj et al.^96^ explored the influence of exercise timing (i.e. the period of the day) on the skeletal muscle proteome in patients with T2DM. Patients were recruited to a randomized crossover trial where they performed two weeks of high‐intensity interval training either in the morning at 8 AM or early evening at 4.45 PM. Interestingly, the authors noted that AM exercise initiated a reduction in skeletal muscle mitochondrial complex III, responsible for transferring electrons from ubiquinol to cytochrome C within the electron transfer chain, and an increase in lipoprotein content, indicating a time‐differential training adaptation to skeletal muscle lipid availability. In addition, they observed training hour‐dependent changes in skeletal muscle structure and lactate‐independent pH regulation through increases in proteins involved in bicarbonate transport, extracellular space, collagen, and exosomes in AM training (Figure 7). These results led the authors to conclude that oxidative capacity in T2DM patients may represent a diurnal component that could help prescribe optimal training strategies in this population. Finally, two publications involved the study of muscle protein signatures in ageing, exercising populations.^112^, ^118^ It is well‐accepted that skeletal muscle mass declines with ageing but exciting research has shown that skeletal muscle performance can be preserved through exercise.^121^ Muscle proteome analysis by label‐free quantitative protein profiling revealed that following 24 h of running, only four of the 429 proteins identified in masters athletes were different from those in a younger cohort.^112^ Interestingly, another ageing study performed saliva analysis to investigate the effects of combined chronic resistance training and submaximal intensity aerobic exercise on protein expression in older exercising individuals.^118^ In this study, 93 of 317 identified proteins were found to be exclusive to exercising individuals, with increased expression of immune response proteins such as lactotransferrin (bactericidal), alpha‐amylase 1, S100‐A8 (antimicrobial), S100‐A9, lactoperoxidase (antimicrobial), and galectin‐3 binding protein (virus and anti‐tumour immune response).^118^

Although proteomics was not as commonly applied in 2022 as other MS‐based techniques, it still remains a useful approach within the SES analytical toolbox. Proteomics has shown to be particularly beneficial for exercise‐based research where tissue‐specific (i.e. muscle) responses are of interest. As with all *‘omics’* methodologies, challenges arise in the biological interpretation of modifications in protein content/quantity. For example, identifying a specific or series of proteins changing across a study investigation is an interesting initial first step; however, the capacity to understand the specificity and/or direct mechanistic action of that protein(s) becomes a far more difficult task. Nonetheless, the opportunity for SES researchers to apply proteomics methods to their research should not be undervalued, with the potential opportunity to identify novel biological mechanisms and/or associations related to an exercise stimulus.

### 3.5 Isotope ratio and elemental MS

IR and elemental MS methods focus on the measurement of stable isotopes or individual elements, either as a way of comparing the levels of incorporated stable isotopes (e.g.13C vs, 12C) or the identification/quantitation of specific element profiles (e.g. metallic ions), respectively. In order to perform these experiments, common protocols include the use of traditional GC‐MS (to identify mass shifts in molecular ions), inductively coupled plasma (ICP)‐MS (to measure element ions, often metallic in nature), and dedicated isotope ratio mass spectrometers (a modernized version of the traditional magnetic sector instrument) with or without pre‐coupling to a chromatographic separation method. The latter instruments are required for the individual measurement of stable isotopes of hydrogen and oxygen which are not compatible with ICP‐MS instruments. In recent years, the use of stable isotope measurements in SES research has been predominantly related to protein turnover (especially within the skeletal muscle), substrate utilization, body water turnover,^11^, ^122^ and in uses to help detect exogenous administration of dopants such as steroid hormones.^123^ Over the past year, a total of 18 studies have been published using IR/elemental approaches in SES. These studies varied across the investigation of carbohydrate (CHO) utilization/oxidation,^20^, ^113^, ^124^, ^125^, ^126^, ^127^, ^128^ protein synthesis/turnover,^50^, ^64^, ^129^, ^130^, ^131^, ^132^ mineral absorption/status,^133^, ^134^, ^135^ energy expenditure,^136^ and environmental exposure.^137^

#### 3.5.1 Carbohydrate utilization/oxidation

**FIGURE 8:**
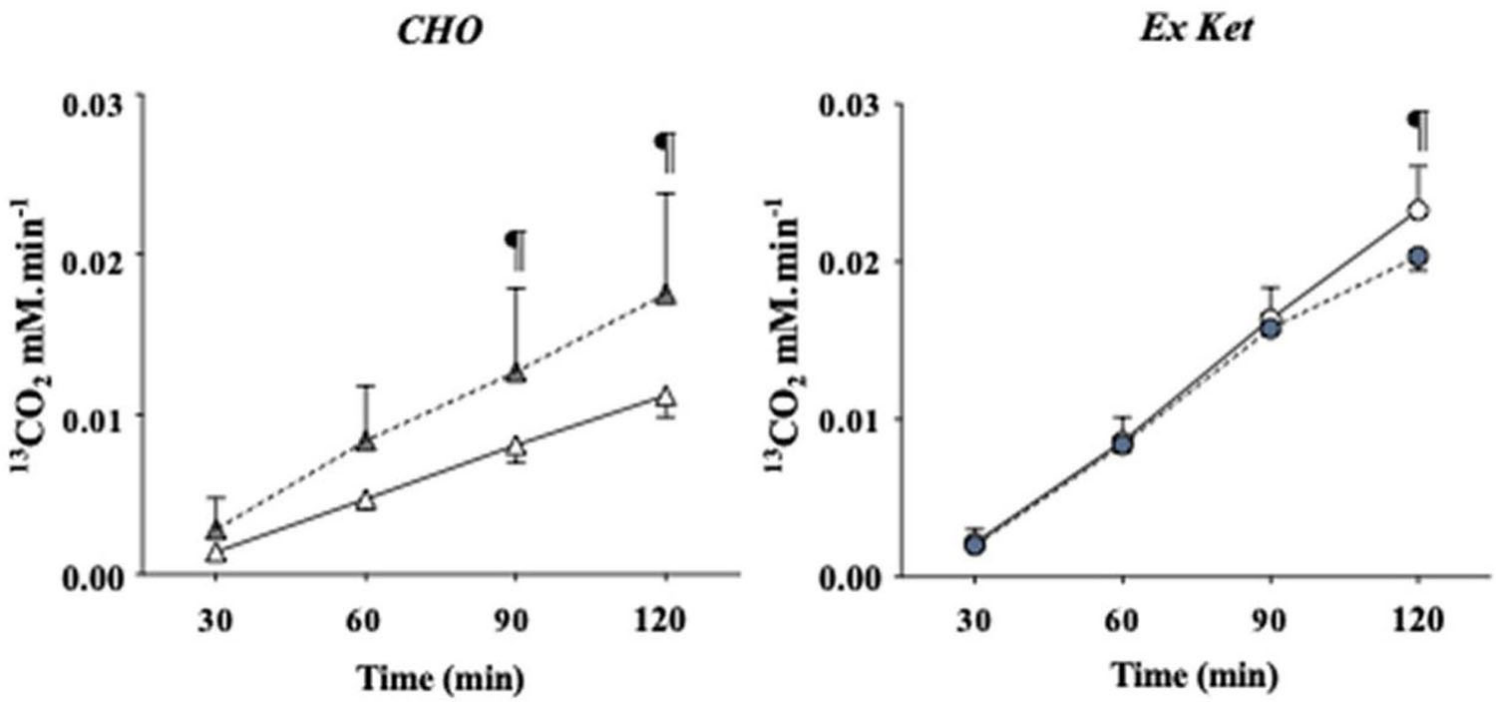
Exhaled ^13^C content following ingestion of a 75 g oral glucose bolus (relative enrichment ^13^C_6_ ∼0.67%) prior to (open markers) and following (closed markers) an 11‐day high carbohydrate diet (CHO) or a normal diet supplemented with a ketone monoester (Ex Ket). Adapted with permission from Dearlove et al.^113^ (Copyright 2022 The Authors, CC‐BY). https://creativecommons.org/licenses/by/4.0/. (¶) Denotes a significant difference between pre‐ and post‐intervention.

IRMS methods provide a robust way to assess CHO oxidation and have, therefore, found themselves popularly applied to SES scenarios. Hearris et al.^125^ used this approach in order to assess the impact of CHO delivery form on exogenous CHO oxidation, gastrointestinal discomfort, and exercise capacity. Nine trained males ingested 120 g/h of CHO (^13^C_6_‐glucose and ^13^C_6_‐fructose enriched) as a fluid, semisolid gel, solid jelly chew, or as a mixed approach whilst completing 3 h of moderate‐intensity cycling exercise (at 95% lactate threshold), followed by an exercise capacity test at 150% lactate threshold. The results demonstrated that neither peak exogenous CHO oxidation, oxidation efficiency, gastrointestinal discomfort, nor exercise capacity was different between the ingestion forms. The authors highlighted this study as the first occasion in which exogenous CHO oxidation has been assessed using IRMS techniques across different feeding strategies. In contrast, Rowe et al.^126^ observed that ingesting CHO as a hydrogel form improved endurance performance (time‐to‐exhaustion test), exogenous CHO oxidation (as assessed by ^13^C:^12^C ratio in plasma and exhaled breath), and gastrointestinal symptoms when compared to a non‐hydrogel based, standard CHO drink. In an alternative approach to the assessment of exogenous labelled glucose oxidation, Dearlove and colleagues^113^ assessed the impact of supplementing with a ketone monoester (Ex Ket) on exercise performance and exogenous CHO oxidation. The results, generated by IRMS analysis of exhaled ^13^C content, demonstrated that 11 days of supplementation with the ketone monoester moderately reduced exogenous CHO oxidation at 120‐min following a 75 g oral glucose bolus (approx. 0.67% relative enrichment of ^13^C_6_). This was in contrast to a high‐CHO diet group which showed endogenous CHO oxidation to increase at 90‐ and 120‐min post glucose bolus following the dietary intervention (Figure 8). Exercise performance was not different between CHO and Ex Ket groups following the supplementation period; however, a group of participants who were prescribed a high‐fat diet to induce a ketogenic state demonstrated a ca. 65% decrease in time‐to‐exhaustion data.

#### 3.5.2 Protein synthesis/turnover

When considering the use of IRMS to assess protein synthesis, two studies completed a deuterium oxide (D_2_O) bolus experiment to calculate the fractional synthesis rate (FSR) by assessing the incorporation of ^2^H labelled alanine into the muscular proteins, with enrichment of body water used as precursor labelling.^129^, ^130^ Both studies utilized a GC‐pyrolysis‐IRMS methodology which involves the separation of amino acids using GC, followed by pyrolysis of the compounds (high‐temperature heating to thermally decompose the compounds) and measurement of the ^2^H content. In the first study, Crossland et al.^129^ investigated the impact of four and eight weeks of concentric or eccentric training on patellar tendon protein synthesis in young and old men. They noted that four weeks of either exercise modality was capable of increasing FSR, returning to rates comparable to the control group at the eight‐week timepoint. Whilst differences between training formats were not noted, the capacity for eccentric exercise to initiate a similar response in protein synthesis to concentric exercise could provide utility in increasing adherence to training in older populations through its reduced time and metabolic commitments. Finally, Davies et al.^130^ examined the impact of fava bean protein (FBP) on post‐exercise myofibrillar FSR. Participants were supplemented with FBP and a placebo in a crossover design and completed a bout of single‐leg knee extensor resistance exercise. It was shown that exercise increased FSR; however, no influence was observed at rest or exercise for FBP ingestion, indicating that it does not have a positive impact on protein synthesis in young, recreationally active adults.

#### 3.5.3 Mineral absorption/status

Regular exposure to high levels of intense training over a prolonged period predisposes athletic populations to low energy availability, leading to aspects of gastrointestinal dysfunction which affect nutrient absorption, and therefore general nutrient availability.^138^ Three studies assessed nutrient status in exercise populations using ICP‐MS, with one investigating both extracellular (serum, plasma and urine) and intracellular (erythrocytes and platelets) zinc concentrations.^134^ In this study, the authors completed a cross‐sectional assessment of young men (∼18–20 years old) who were either semi‐professional youth soccer players (10 h of structured training per week) or age‐matched non‐active individuals. Despite no differences in dietary zinc intake across groups, soccer players were identified to have a higher zinc concentration in the plasma but a lower concentration in erythrocytes. The authors concluded that increased plasma zinc levels may have been attributable to muscle cell leakage from exercise‐induced damage, with an additional possibility of exercise‐induced hemolysis adding to increased zinc plasma but decreased levels within red blood cell content. Barney et al.^133^ studied the iron status of female and male collegiate cross‐country runners. The participants were provided with a stable iron isotope (^54^Fe) via a beverage given two hours after long‐distance running training or following a resting period. Blood samples were collected 1 h post‐ingestion and red blood cells were isolated for fractional iron absorption levels. The protocol identified that dietary iron absorption was decreased by 36% at 3 h following exercise versus the resting protocol. This supports the notion that exercise reduces iron absorption capacity which may lead to deficiencies in exercising populations. Interestingly, there was a difference in post‐exercise hepcidin concentration between sexes (a protein involved in iron absorption) but no difference was observed for overall fractional iron absorption, suggesting that exercise itself may not be a driver for increased risk of iron deficiency observed in female athletes.

Importantly, as it stands, we are not aware of any documentation that outlines the minimum expected reporting information for IRMS and elemental analyses. We, therefore, recommend that experts within these fields work together in order to generate this to aid in the validity of future research experiments.

## 4 FUTURE CONSIDERATIONS FOR MS IN SES

Whilst the application of MS is undoubtedly increasing year‐on‐year, it is considered by the authors that there are still many aspects that need addressing within SES research. First, it is clear from the research presented in this annual review that there is a keen interest in the application of MS to perform targeted, most often quantitative, assays using GC/LC‐MS, IRMS and ICP‐MS. However, it must be noted that, in striking reality, the validity of much of the data present using quantitative MS in SES must be considered with caution. From the studies using targeted methodologies, only one study included validation data, one used a commercial assay, and 11 (16%) gave direct reference to a manuscript which detailed sufficient method validation to warrant robust quantitative data interpretation. For this level of validation, the authors must demonstrate that the method is precise and accurate, and provide information for recovery, matrix effects, (lack of) carryover, limits of quantitation and detection, linearity, and, ideally, analyte stability (i.e. over time or following freeze/thaw cycles). In general, method validation exercises such as these follow the bioanalytical guidelines set by the US Food and Drug Administration^139^ or the European Medicines Agency.^140^ It is startling that approximately two‐thirds (66%) of all relevant studies in this review provided no, or entirely insufficient, data for method validation. Perhaps even more alarmingly, ten studies (15%) cited previous papers as a reference to the validity of their analysis which, after additional research, linked back to publications that had, again, insufficient or a complete lack of data regarding method validation. As many of the studies completed in SES rely on quantitative comparisons of analytes, it is imperative that the understanding and uptake of fully validated methods, with demonstrable evidence, is achieved.

Historically, SES research has been focused on the study of male participants, seemingly due to the consideration that the lack of the menstrual hormone cycle will create fewer counter interactions with aspects of physiology and motivation related to exercise performance and sport. On this note, a recent publication demonstrated that from 669 studies published between 2017 and 2021 in six major sports medicine journals, only ca. 30% of studies included females as participants alongside males.^141^ In addition, only ca. 9% of studies isolated females for independent study, with a staggering ca. 71% of studies including male‐only participants. Another study from 2014 also noted that when both males and females were included in the investigations (from 1382 studies in three leading sports medicine journals), the studied cohorts included an average of only 35%–37% contribution from female participants.^142^ In 2022, 43 studies across all topics and approaches included within this review exclusively recruited female participants or explicitly reported an equal or predominantly (≥50%) female study cohort, representing approximately 46% included within this manuscript, albeit noted that much of the female dominant research is more clinically focused, showing a slightly increased trend to that shown in the previously mentioned review articles. The studies identified to include ≥50% of female participants can be identified in the Supporting Information. These data demonstrate that an increase in the use of females in SES research is required; however, this is caveated with the importance of accurate and precise measurement of female hormonal status. For this, sex steroid hormones (notably estradiol and progesterone) are commonly measured in female participants recruited to exercise‐based studies. This is due to the potential effects that these hormones may have on exercise performance as their levels fluctuate across the menstrual cycle.^144^ Traditionally, and still common today, these molecules are measured using an immunoassay approach. However, it is well known that the major limitation of immunoassay measurements of steroid hormones is the interference caused by structurally similar compounds and their metabolites,^145^ with significant similarities between steroid hormones due to them all being derived from cholesterol. This limitation is also compounded by issues relating to sensitivity and standardization across laboratories.^146^ MS‐based assays have the capacity to overcome these limitations, so much so that it is now recommended by clinical journals that measurements for sex steroids should be conducted using MS, with specific reference to avoid immunoassay methods.^147^ However, and importantly, the use of MS‐based methods for the measurement of female‐relevant sex hormones is few and far between within the SES field. This identifies an area where SES can benefit greatly from the increased inclusion of analytical chemistry within its ranks, calling for a genuine requirement to increase the presence of MS in SES‐based laboratories/departments and the technical capacity of SES researchers to be able to perform these assays to provide the most reliable, reproducible, and standardizable measurements for female hormone investigation. From the defined search functions, only one publication in 2022 relating to female sex hormone analysis using MS in an exercise/sport‐based protocol was identified. This study by Dalgaard et al.^36^ observed a reduction in intramuscular estradiol, testosterone, and dehydroepiandrosterone in postmenopausal women following a 12‐week resistance training intervention, but with no changes seen in an acute exercise setting. These findings were seen irrespective of estrogen therapy status. In addition, they noted that low levels of serum follicle‐stimulating hormone (FSH) and luteinizing hormone (LH) were associated with high muscle mass at baseline, with declines in serum FSH levels observed following the increase of muscle mass brought about by the training program. From these results, the authors concluded that serum FSH and LH were improved predictors of muscle mass compared to other circulating sex steroid hormones. Importantly, it must be noted that this study provided no details regarding the MS method employed making it impossible for readers to replicate the work, although the authors did note that some analyses were completed in an ISO‐accredited clinical laboratory.

In addition to participant characteristics, it is also important to consider the number of individuals recruited to participate in these research studies. Due to the large‐data, wide‐scope approach performed across the *‘omics’* topics, it would be preferential (for data investigation purposes) that these studies were able to generate large participant datasets. However, in 2022 there was little overall difference seen in the numbers recruited between targeted [median (range); 31 (5–268,964)] and non‐targeted studies [32 (5–105)], albeit 11 targeted studies contained more participants than the largest of the non‐targeted experiments. This is perhaps not surprising as the application of non‐targeted methodologies is more time‐consuming and often more costly. Nonetheless, there is a need for these studies to recruit substantial participant numbers in order to provide sufficient power for data investigations. Details of participant numbers recruited to each individual study are included within the Supporting Information.

**FIGURE 9:**
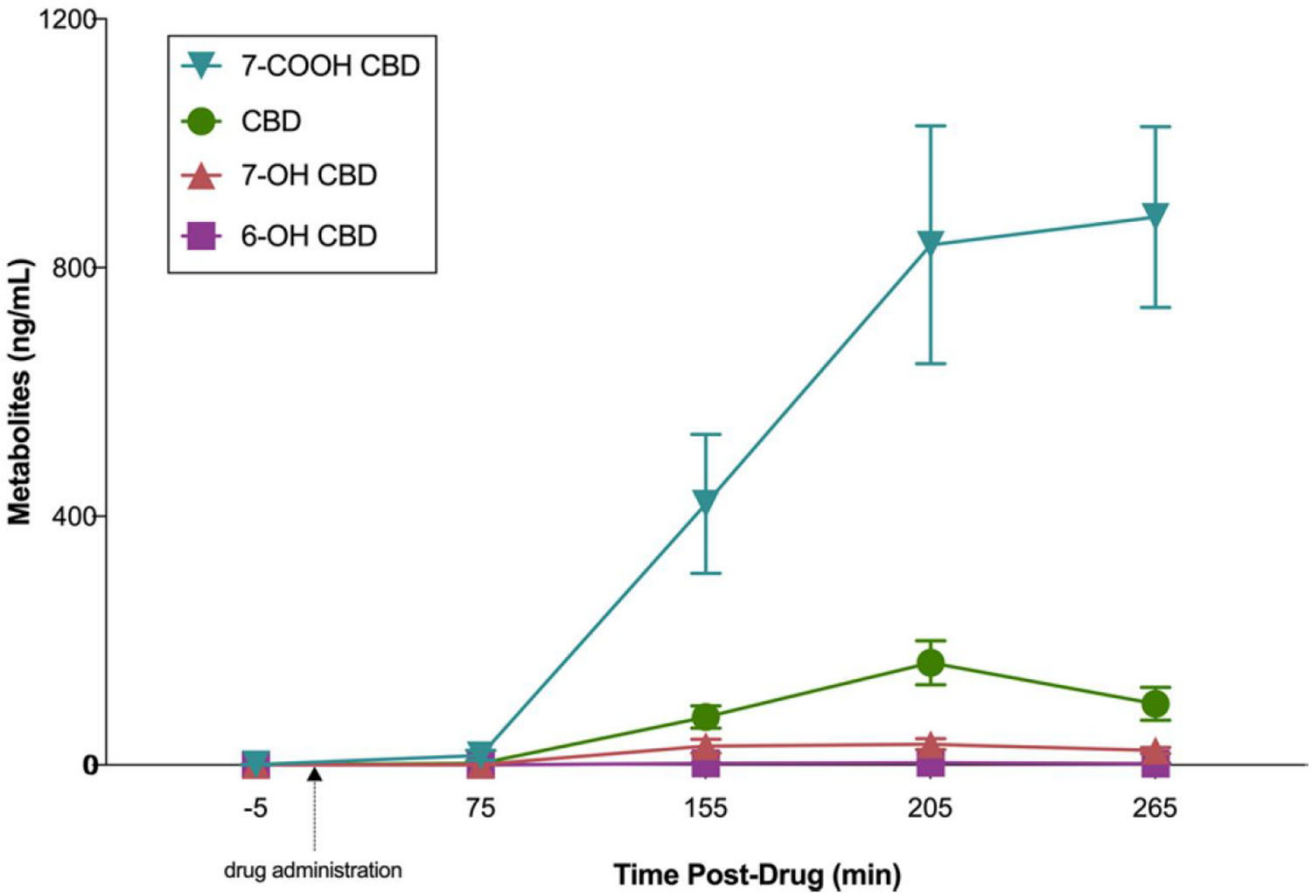
Measurements of circulating cannabidiol (CBD), and its carboxy (7‐COOH CBD) and hydroxy (7‐OH CBD, 6‐OH CBD) metabolites following a 300 mg oral dose of CBD across 5 min (baseline), 75 min (pre‐submaximal exercise), 155 min (post‐submaximal exercise), 205 min (post‐maximal exercise), and 265 min (1‐h post‐maximal exercise). Adapted with permission from Sahinovic et al.^32^ (Copyright 2022 The Authors, CC‐BY). https://creativecommons.org/licenses/by/4.0/.

Finally, an aspect that has not been heavily discussed during this review is the use of MS to assess the bioavailability and pharmacokinetics/pharmacodynamics (PKPD) of exercise/sports‐based nutritional supplementation strategies. Although it is not the purpose of this review to identify studies which do not apply MS techniques; it is, in general, our experience that many of the nutritional intervention studies performed in SES do not assess the impact of the supplement on circulating levels (i.e. using MS or by other means to quantitate the parent or metabolite forms). This is perhaps due to the fact that the authors cannot perform the analysis with the equipment available to them (reinforcing the point of increasing accessibility to MS in SES stated above), or that they do not consider the bioavailability/PKPD as an important factor for the interpretation of the study results. We would argue that these data are extremely important, especially if assessing an acute impact on exercise outcomes. This consideration links to a recent surge in the interest in cannabidiol (CBD) as an ergogenic aid in exercise performance and/or recovery.^148^, ^149^ As CBD is not endogenous to the human system, it is important to understand its absorption profiles, bioavailability and distribution, the kinetics of metabolism, and excretion rates, especially as these could be affected by individual‐ and environmental‐specific cofactors. For this reason, it was encouraging to see the use of MS to measure both CBD and its hydroxy/carboxy metabolites in an exercise physiology study published in 2022.^32^ In this study, ten participants completed a randomized crossover trial where they were administered an acute dose of 300 mg CBD or placebo. The researchers investigated the impact of oral CBD ingestion on features of submaximal and maximal exercise. Supplementation with CBD showed a rise in CBD and 7‐carboxy‐CBD ca. four‐fold greater than CBD from 155 min post‐ingestion, remaining above pre‐ingestion levels until the end of the sampling period (255 min post‐ingestion) (Figure 9). Although effect sizes demonstrated uncertain differences between trials, it was observed that possible increases in oxygen uptake, carbon dioxide production, and blood lactate were seen in the CBD trial during the submaximal run, with possible increases in VO_2peak_, VCO_2__peak_, ventilatory threshold, and time‐to‐exhaustion. In addition to the measurement of CBD bioavailability, the use of MS for the measurement of CBD‐containing products is an important consideration for the quantitation of CBD, along with tetrahydrocannabinol and other cannabinoids which are currently prohibited for use during sporting competition cycles. As CBD is the only cannabinoid not included on WADA's *Prohibited List*, its use as an ergogenic aid is fully warranted by athletes; however, the efficacy of the product (i.e. are CBD levels really as advertised) and the presence of prohibited cannabinoids, remains an important (and contentious) aspect when considering the availability of products to consumers in the exercise science market.^150^

## 5 SUMMARY AND OUTLOOK

It is clear from the articles included in this review that there is an appetite for the use of MS in SES‐based research investigations. The current trends demonstrate that a targeted, mostly quantitative, approach is the most used within the field, which is to be expected due to the traditional nature of SES chasing answers to hypothesis‐driven research questions. These targeted applications are generally centred around the measurement of endogenous or exogenous metabolites, but can also be extended to incorporate the direct analysis of metabolic enrichment and mineral status through IRMS and elemental MS methods, respectively. Interestingly, although not all SES research is based on (sub‐)elite athlete populations, it may be that the field of *Mass Sportrometry* will require the development and establishment of athlete‐specific reference values/intervals, especially as these may differ from the general population through the presence of a heightened training state. There has also been a keen interest in the use of *‘omics’* to perform hypothesis‐generating research studies, with the applications of metabolomics and proteomics the most popular of these methodologies. Lipidomics has shown to be less common in the SES field at this time, but this is reflective of the use of lipidomics across the majority of research topics. It has shown a slow, but steady, growth in clinical research that will undoubtedly translate to SES studies as the field develops and more researchers become familiar with the methods and their interpretations. For instance, lipidomics offers great potential for research in the physical activity space, especially within overweight/obese populations where lipid species may be important drivers of disease.

It is apparent that, overall, the use of MS is by no means commonplace in SES at this time. Although the interest is demonstrable from the 93 publications cited, this is only a small proportion of SES‐based research that will have been published across 2022. That is not to say that MS must be included in these research studies where it is not required, but the consideration for its use to expand, and possibly improve, the analytical options within the field should be taken with open arms and confidence. Of course, at this point in time, the use of MS by SES researchers predominantly relies on local, national, and international collaborations with analytical scientists; however, it is the view of the authors that the integration of MS technologies into SES laboratories/departments is a genuine opportunity to exploit the benefits of analytical chemistry in exercise/sports‐based research. Naturally, this requires investment in both finances and time (and certainly personnel and infrastructure!) in these early stages of transition, but we believe the benefits are befitting of the efforts required to achieve these goals. What is more, in order to improve the application of analytical science in SES we should be expanding the teaching given to our SES students who will make up a proportion of the future research base. Considering MS is a stalwart of the anti‐doping laboratory, it seems appropriate that our students should understand the fundamental principles of its workings, along with basic knowledge of analytical science including vital aspects of analysis including accuracy and precision. Only by instilling these understandings into the current, and future, generation of SES scientists will we truly transform the analytical expertise within the field.

Overall, it is exciting to see the increasing adoption of MS in SES, with a wide array of applications demonstrated in this review. We expect the number, and quality, of MS‐based assays in SES to increase gradually over time, and look forward with anticipation to seeing what 2023 will bring.

## AUTHOR CONTRIBUTIONS

Marilyn LY Ong: Writing—original draft (equal), review and editing. Christopher G Green: Writing—original draft, review and editing. Samantha N Rowland: Writing—original draft, review and editing. Liam M Heaney: Conceptualization (lead). Writing—original draft (equal), review and editing (lead).

## CONFLICT OF INTEREST STATEMENT

The authors declare no conflicts of interest.

## FUNDING INFORMATION

Marilyn LY Ong is supported by the Ministry of Higher Education Malaysia and Universiti Sains Malaysia Post‐Doctoral Fellowship. Christopher G Green is supported by a doctoral research scholarship administered by the School of Sport, Exercise and Health Sciences and the Doctoral College at Loughborough University. Samantha N Rowland is supported by external research funding awarded to Liam M Heaney from ADM Biopolis. The funders played no role in the production of this manuscript.

## Supporting information



Supporting Information

## Data Availability

Data sharing is not applicable to this article as no new data were created.

## References

[1] Heaney LM . Applying mass spectrometry‐based assays to explore gut microbial metabolism and associations with disease. Clin Chem Lab Med. 2020;58(5):719‐732. doi:10.1515/cclm-2019-0974 31639103

[2] Seger C , Salzmann L . After another decade: LC–MS/MS became routine in clinical diagnostics. Clin Biochem. 2020;82:2‐11. doi:10.1016/j.clinbiochem.2020.03.004 32188572

[3] Kussmann M , Affolter M , Nagy K , Holst B , Fay LB . Mass spectrometry in nutrition: understanding dietary health effects at the molecular level. Mass Spectrom Rev. 2007;26(6):727‐750. doi:10.1002/mas.20147 17654467

[4] Feng X , Liu X , Luo Q , Liu B‐F . Mass spectrometry in systems biology: an overview. Mass Spectrom Rev. 2008;27(6):635‐660. doi:10.1002/mas.20182 18636545

[5] Thevis M , Kuuranne T , Geyer H . Annual banned‐substance review—Analytical approaches in human sports drug testing 2021/2022. Drug Test Anal. doi:10.1002/dta.3408. Published online 2022. 29149502

[6] Eugster PJ , Bourdillon N , Vocat C , et al. Kinetics of neuropeptide Y, catecholamines, and physiological responses during moderate and heavy intensity exercises. Neuropeptides. 2022;92:102232. doi:10.1016/j.npep.2022.102232 35180646

[7] Pedersen BK , Saltin B . Exercise as medicine ‐ evidence for prescribing exercise as therapy in 26 different chronic diseases. Scand J Med Sci Sports. 2015;25:1‐72. doi:10.1111/sms.12581 26606383

[8] American College of Sports Medicine . ACSM's Guidelines for Exercise Testing and Prescription. 11th ed. Wolters Kluwer; 2022. 10.1249/JSR.0b013e31829a68cf 23851406

[9] Allgayer H , Owen RW , Nair J , et al. Short‐term moderate exercise programs reduce oxidative DNA damage as determined by high‐performance liquid chromatography‐electrospray ionization‐mass spectrometry in patients with colorectal carcinoma following primary treatment. Scand J Gastroenterol. 2008;43(8):971‐978. doi:10.1080/00365520701766111 18609189

[10] I. Bachini F, Pereira D , Santos R , et al. Creatine and creatinine quantification in olympic athletes: dried blood spot analysis pilot study. Biol Sport. 2022;39(3):745‐749. doi:10.5114/biolsport.2022.108701 35959322 PMC9331328

[11] Cataldi D , Bennett JP , Quon BK , et al. Agreement and precision of deuterium dilution for total body water and multicompartment body composition assessment in collegiate athletes. J Nutr. 2022;152(9):2048‐2059. doi:10.1093/jn/nxac116 35665820

[12] Gomez‐Gomez A , Olesti E , Montero‐San‐Martin B , et al. Determination of up to twenty carboxylic acid containing compounds in clinically relevant matrices by o‐benzylhydroxylamine derivatization and liquid chromatography‐tandem mass spectrometry. J Pharm Biomed Anal. 2022;208:114450. doi:10.1016/j.jpba.2021.114450 34798391

[13] Martínez Brito D , Leogrande P , Donati F , de la Torre X , Botrè F . Quantification of thyroid hormones and analogs by liquid chromatography coupled to mass spectrometry. Preliminary results in athletes and non‐athletes serum samples. Drug Test Anal. 2022;14(8):1438‐1450. doi:10.1002/dta.3269 35368147

[14] Mashal MS , Nalin M , Bevalot F , Sallet P , Guitton J , Machon C . Simultaneous quantification of 19 nonsteroidal anti‐inflammatory drugs in oral fluid by liquid chromatography‐high resolution mass spectrometry: application on ultratrail runner's oral fluid. Drug Test Anal. 2022;14(4):701‐712. doi:10.1002/dta.3216 34989157

[15] van den Broek J , Mochalski P , Königstein K , et al. Selective monitoring of breath isoprene by a portable detector during exercise and at rest. Sensors Actuators B Chem. 2022;357:131444. doi:10.1016/j.snb.2022.131444

[16] Forteza F , Bourdeau‐Julien I , Nguyen GQ , et al. Influence of diet on acute endocannabinoidome mediator levels post exercise in active women, a crossover randomized study. *Sci Rep* . 2022;12(1):8568. doi:10.1038/s41598-022-10757-0 PMC9122896 35595747

[17] Tataka Y , Haramura M , Hamada Y , et al. Effects of oral cystine and glutamine on exercise‐induced changes in gastrointestinal permeability and damage markers in young men. Eur J Nutr. 2022;61(5):2331‐2339. doi:10.1007/s00394-022-02806-1 35106632 PMC9279189

[18] Singh A , D'Amico D , Andreux PA , et al. Urolithin A improves muscle strength, exercise performance, and biomarkers of mitochondrial health in a randomized trial in middle‐aged adults. Cell Reports Med. 2022;3(5):100633. doi:10.1016/j.xcrm.2022.100633 PMC9133463 35584623

[19] Jaramillo‐Morales J , Korucu B , Pike MM , et al. Effects of caloric restriction and aerobic exercise on circulating cell‐free mitochondrial DNA in patients with moderate to severe chronic kidney disease. Am J Physiol Physiol. 2022;322(1):F68‐F75. doi:10.1152/ajprenal.00270.2021 PMC8742721 34843657

[20] Løkken N , Storgaard JH , Revsbech KL , et al. No effect of oral ketone ester supplementation on exercise capacity in patients with McArdle disease and healthy controls: a randomized placebo‐controlled cross‐over study. J Inherit Metab Dis. 2022;45(3):502‐516. doi:10.1002/jimd.12484 35150142 PMC9304134

[21] Chaouachi M , Gautier S , Carnot Y , et al. Spirulina supplementation prevents exercise‐induced lipid peroxidation, inflammation and skeletal muscle damage in elite rugby players. J Hum Nutr Diet. 2022;35(6):1151‐1163. doi:10.1111/jhn.13014 35394687

[22] Revuelta Iniesta R, Causer AJ , Arregui‐Fresneda I , et al. The impact of plasma 25‐hydroxyvitamin D on pulmonary function and exercise physiology in cystic fibrosis: a multicentre retrospective study. J Hum Nutr Diet. 2022;35(2):363‐375. doi:10.1111/jhn.12906 33908093

[23] Wangdi JT , O'Leary MF , Kelly VG , et al. Tart cherry supplement enhances skeletal muscle glutathione peroxidase expression and functional recovery after muscle damage. Med Sci Sport Exerc. 2022;54(4):609‐621. doi:10.1249/MSS.0000000000002827 34772901

[24] Liu S , D'Amico D , Shankland E , et al. Effect of urolithin a supplementation on muscle endurance and mitochondrial health in older adults. JAMA Netw Open. 2022;5(1):e2144279. doi:10.1001/jamanetworkopen.2021.44279 35050355 PMC8777576

[25] Li K , Schön M , Naviaux JC , et al. Cerebrospinal fluid and plasma metabolomics of acute endurance exercise. FASEB J. 2022;36(7):e22408. doi:10.1096/fj.202200509R 35713567

[26] Wang R , Wu X , Lin K , et al. Plasma metabolomics reveals β‐glucan improves muscle strength and exercise capacity in athletes. Metabolites. 2022;12(10):988. doi:10.3390/metabo12100988 36295890 PMC9607031

[27] Gillies NA , Franzke B , Wessner B , et al. Nutritional supplementation alters associations between one‐carbon metabolites and cardiometabolic risk profiles in older adults: a secondary analysis of the Vienna Active Ageing Study. Eur J Nutr. 2022;61(1):169‐182. doi:10.1007/s00394-021-02607-y 34240265 PMC8783863

[28] Jurado‐Fasoli L , Yang W , Kohler I , et al. Effect of different exercise training modalities on fasting levels of oxylipins and endocannabinoids in middle‐aged sedentary adults: a randomized controlled trial. Int J Sport Nutr Exerc Metab. 2022;32(4):275‐284. doi:10.1123/ijsnem.2021-0332 35339112

[29] Kosaki K , Kumamoto S , Tokinoya K , et al. Xanthine oxidoreductase activity in marathon runners: potential implications for marathon‐induced acute kidney injury. J Appl Physiol. 2022;133(1):1‐10. doi:10.1152/japplphysiol.00669.2021 35608201

[30] Li VL , He Y , Contrepois K , et al. An exercise‐inducible metabolite that suppresses feeding and obesity. Nature. 2022;606(7915):785‐790. doi:10.1038/s41586-022-04828-5 35705806 PMC9767481

[31] Warensjö Lemming E, Petrelius Sipinen J, Nyberg G , Moraeus L , Lindroos AK . Vitamin D status and associations with diet, objectively measured physical activity patterns and background characteristics among adolescents in a representative national cross‐sectional survey. Public Health Nutr. 2022;25(6):1427‐1437. doi:10.1017/S1368980022000222 35067271 PMC9991641

[32] Sahinovic A , Irwin C , Doohan PT , et al. Effects of cannabidiol on exercise physiology and bioenergetics: a randomised controlled pilot trial. Sport Med Open. 2022;8(1):27. doi:10.1186/s40798-022-00417-y PMC8891421 35235092

[33] Strom CJ , McDonald SM , Remchak M‐M , et al. The influence of maternal aerobic exercise, blood DHA and EPA concentrations on maternal lipid profiles. Int J Environ Res Public Health. 2022;19(6):3550. doi:10.3390/ijerph19063550 35329235 PMC8949039

[34] Tsikas D , Maassen N , Thorns A , et al. Short‐Term supplementation of sodium nitrate vs. sodium chloride increases homoarginine synthesis in young men independent of exercise. Int J Mol Sci. 2022;23(18):10649. doi:10.3390/ijms231810649 36142560 PMC9504822

[35] Grillet P‐E , Badiou S , Lambert K , et al. Biomarkers of redox balance adjusted to exercise intensity as a useful tool to identify patients at risk of muscle disease through exercise test. Nutrients. 2022;14(9):1886. doi:10.3390/nu14091886 35565853 PMC9105000

[36] Dalgaard LB , Oxfeldt M , Dam TV , Hansen M . Intramuscular sex steroid hormones are reduced after resistance training in postmenopausal women, but not affected by estrogen therapy. Steroids. 2022;186:109087. doi:10.1016/j.steroids.2022.109087 35809683

[37] Carswell AT , Jackson S , Swinton P , et al. Vitamin D metabolites are associated with physical performance in young healthy adults. Med Sci Sport Exerc. 2022;54(11):1982‐1989. doi:10.1249/MSS.0000000000002987 35766614

[38] Jung C‐Y , Chun HS , Lee M , et al. Exercise reduces the risk of chronic kidney disease in individuals with nonalcoholic fatty liver disease: a nationwide cohort study. Diabetes Metab. 2022;48(5):101362. doi:10.1016/j.diabet.2022.101362 35660527

[39] Allman BR , Spray BJ , Lan RS , Andres A , Børsheim E . Circulating long‐chain acylcarnitine concentrations are not affected by exercise training in pregnant women with obesity. J Appl Physiol. 2022;132(2):470‐476. doi:10.1152/japplphysiol.00712.2021 34989648 PMC8816616

[40] Li X , Li K , Zhu Z , et al. Exercise regulates the metabolic homeostasis of methamphetamine dependence. Metabolites. 2022;12(7):606. doi:10.3390/metabo12070606 35888730 PMC9323070

[41] Zeng G , Zhang Q , Wang X , Wu K‐H . The relationship between multiple perfluoroalkyl substances and cardiorespiratory fitness in male adolescents. Environ Sci Pollut Res. 2022. doi:10.1007/s11356-022-19685-y 35288850

[42] Zhang Q , Xu Q , Tian H , Chu Y , Qiu J , Sun M . Serum and diet long‐chain omega‐3 fatty acid nutritional status in Chinese elite athletes. Lipids. 2023;58(1):33‐40. doi:10.1002/lipd.12362 36271748

[43] Gagesch M , Wieczorek M , Vellas B , et al. Effects of Vitamin D, omega‐3 fatty acids and a home exercise program on prevention of pre‐frailty in older adults: the DO‐HEALTH randomized clinical trial. J Frailty Aging. doi:10.14283/jfa.2022.48. Published online 2022. 36629088

[44] Luk AWS , Mitchell L , Koay YC , et al. Intersection of diet and exercise with the gut microbiome and circulating metabolites in male bodybuilders: a pilot study. *Metabolites*. 2022;12(10):911. doi:10.3390/metabo12100911 PMC9608465 36295813

[45] Maalouf NM , Chhabra A , Zafereo J , et al. Androgen deprivation therapy differentially impacts bone and muscle in the short term in physically active men with prostate cancer. JBMR Plus. 2022;6(1):e10573. doi:10.1002/jbm4.10573 35079681 PMC8770993

[46] Machek SB , Zawieja EE , Heileson JL , et al. Human serum betaine and associated biomarker concentrations following a 14 day supplemental betaine loading protocol and during a 28 day washout period: a pilot investigation. Nutrients. 2022;14(3):498. doi:10.3390/nu14030498 35276860 PMC8839982

[47] Patten RK , McIlvenna LC , Levinger I , et al. High‐intensity training elicits greater improvements in cardio‐metabolic and reproductive outcomes than moderate‐intensity training in women with polycystic ovary syndrome: a randomized clinical trial. Hum Reprod. 2022;37(5):1018‐1029. doi:10.1093/humrep/deac047 35325125

[48] Perreault M , Mottola MF , Atkinson SA , et al. Individualized high dairy protein + walking program supports bone health in pregnancy: a randomized controlled trial. Am J Clin Nutr. 2022;25:887‐896. doi:10.1093/ajcn/nqac182 35759368

[49] Thams L , Hvid LG , Stounbjerg NG , et al. Vitamin D supplementation and increased dairy protein intake do not affect muscle strength or physical function in healthy 6–8‐year‐old children: the D‐pro randomized trial. Eur J Nutr. 2022;61(7):3613‐3623. doi:10.1007/s00394-022-02912-0 35643873 PMC9146815

[50] Nishimura Y , Jensen M , Bülow J , et al. Co‐ingestion of cluster dextrin carbohydrate does not increase exogenous protein‐derived amino acid release or myofibrillar protein synthesis following a whole‐body resistance exercise in moderately trained younger males: a double‐blinded randomized contr. Eur J Nutr. 2022;61(5):2475‐2491. doi:10.1007/s00394-021-02782-y 35182194 PMC9279228

[51] Bjørnebekk A , Scarth M , Neupane SP , et al. Use of high‐dose androgens is associated with reduced brain derived neurotrophic factor in male weightlifters. Neuroendocrinology. doi:10.1159/000526418. Published online 2022. 35944495

[52] Rodas G , Ferrer E , Reche X , Sanjuan‐Herráez JD , McCall A , Quintás G . A targeted metabolic analysis of football players and its association to player load: comparison between women and men profiles. Front Physiol. 2022;13:923608. doi:10.3389/fphys.2022.923608 36246100 PMC9561103

[53] Withycombe JS , Eldridge R , Jin Y , Gu H , Castellino SM , Sears DD . Metabolites associated with fatigue and physical activity in childhood cancer. Biol Res Nurs. 2022;24(3):350‐361. doi:10.1177/10998004221085029 35466716 PMC9343883

[54] Xu Y , Gao H , Du Z , et al. A new approach for reducing pollutants level: a longitudinal cohort study of physical exercises in young people. BMC Public Health. 2022;22(1):223. doi:10.1186/s12889-022-12621-2 35114971 PMC8812347

[55] Legaard GE , Feineis CS , Johansen MY , et al. Effects of an exercise‐based lifestyle intervention on systemic markers of oxidative stress and advanced glycation endproducts in persons with type 2 diabetes: secondary analysis of a randomised clinical trial. Free Radic Biol Med. 2022;188:328‐336. doi:10.1016/j.freeradbiomed.2022.06.013 35764194

[56] Williams CJ , Torquati L , Li Z , et al. Oligofructose‐enriched inulin intake, gut microbiome characteristics, and the v̇o2 peak response to high‐intensity interval training in healthy inactive adults. J Nutr. 2022;152(3):680‐689. doi:10.1093/jn/nxab426 34910161

[57] Torquati L , Gajanand T , Cox ER , et al. Effects of exercise intensity on gut microbiome composition and function in people with type 2 diabetes. Eur J Sport Sci. doi:10.1080/17461391.2022.2035436. Published online 2022. 35107058

[58] Bernhardt V , Stickford JL , Bhammar DM , Balmain BN , Babb TG . Repeatability of dyspnea measurements during exercise in women with obesity. Respir Physiol Neurobiol. 2022;297:103831. doi:10.1016/j.resp.2021.103831 34922000 PMC11463220

[59] Moriyama S , Ichinose M , Dobashi K , et al. Hypercapnia elicits differential vascular and blood flow responses in the cerebral circulation and active skeletal muscles in exercising humans. Physiol Rep. 2022;10(8):e15274. doi:10.14814/phy2.15274 35466573 PMC9035754

[60] Lake SL , Guadagni V , Kendall KD , et al. Aerobic exercise training in older men and women—Cerebrovascular responses to submaximal exercise: results from the Brain in Motion study. Physiol Rep. 2022;10(4):e15158. doi:10.14814/phy2.15158 35212167 PMC8874289

[61] Tarumi T , Patel NR , Tomoto T , et al. Aerobic exercise training and neurocognitive function in cognitively normal older adults: a one‐year randomized controlled trial. J Intern Med. 2022;292(5):788‐803. doi:10.1111/joim.13534 35713933 PMC9588521

[62] Güntner AT , Weber IC , Schon S , Pratsinis SE , Gerber PA . Monitoring rapid metabolic changes in health and type‐1 diabetes with breath acetone sensors. Sensors Actuators B Chem. 2022;367:132182. doi:10.1016/j.snb.2022.132182

[63] Tomoto T , Verma A , Kostroske K , et al. One‐year aerobic exercise increases cerebral blood flow in cognitively normal older adults. J Cereb Blood Flow Metab. doi:10.1177/0271678X221133861. Published online 2022. PMC9941859 36250505

[64] Kunz HE , Michie KL , Gries KJ , Zhang X , Ryan ZC , Lanza IR . A randomized trial of the effects of dietary n3‐PUFAs on skeletal muscle function and acute exercise response in healthy older adults. Nutrients. 2022;14(17):3537. doi:10.3390/nu14173537 36079794 PMC9459748

[65] Bekesiene S , Smaliukiene R , Vaičaitienė R , et al. Three‐faceted approach to perceived stress: a longitudinal study of stress hormones, personality, and group cohesion in the real‐life setting of compulsory basic military training. Sustainability. 2022;14(3):1046. doi:10.3390/su14031046

[66] Puigarnau S , Fernàndez A , Obis E , et al. Metabolomics reveals that fittest trail runners show a better adaptation of bioenergetic pathways. J Sci Med Sport. 2022;25(5):425‐431. doi:10.1016/j.jsams.2021.12.006 35063356

[67] Zhang R , Naughton DP . Vitamin D in health and disease: current perspectives. Nutr J. 2010;9(1):65. doi:10.1186/1475-2891-9-65 21143872 PMC3019131

[68] Antoniak AE , Greig CA . The effect of combined resistance exercise training and vitamin D 3 supplementation on musculoskeletal health and function in older adults: a systematic review and meta‐analysis. BMJ Open. 2017;7(7):e014619. doi:10.1136/bmjopen-2016-014619 PMC5541589 28729308

[69] Wiciński M , Adamkiewicz D , Adamkiewicz M , et al. Impact of Vitamin D on physical efficiency and exercise performance—a review. Nutrients. 2019;11(11):2826. doi:10.3390/nu11112826 31752277 PMC6893541

[70] Salles J , Chanet A , Giraudet C , et al. 1,25(OH) 2 ‐vitamin D 3 enhances the stimulating effect of leucine and insulin on protein synthesis rate through Akt/PKB and mTOR mediated pathways in murine C2C12 skeletal myotubes. Mol Nutr Food Res. 2013;57(12):2137‐2146. doi:10.1002/mnfr.201300074 23929734

[71] Binkley N , Krueger D , Cowgill CS , et al. Assay variation confounds the diagnosis of hypovitaminosis D: a call for standardization. J Clin Endocrinol Metab. 2004;89(7):3152‐3157. doi:10.1210/jc.2003-031979 15240586

[72] Holick MF . Vitamin D status: measurement, interpretation, and clinical application. Ann Epidemiol. 2009;19(2):73‐78. doi:10.1016/j.annepidem.2007.12.001 18329892 PMC2665033

[73] García‐Villalba R , Giménez‐Bastida JA , Cortés‐Martín A , et al. Urolithins: a comprehensive update on their metabolism, bioactivity, and associated gut microbiota. Mol Nutr Food Res. 2022;66(21):2101019. doi:10.1002/mnfr.202101019 35118817 PMC9787965

[74] D'Amico D , Andreux PA , Valdés P , Singh A , Rinsch C , Auwerx J . Impact of the natural compound urolithin A on health, disease, and aging. Trends Mol Med. 2021;27(7):687‐699. doi:10.1016/j.molmed.2021.04.009 34030963

[75] Koh A , De Vadder F , Kovatcheva‐Datchary P , Bäckhed F . From dietary fiber to host physiology: short‐chain fatty acids as key bacterial metabolites. Cell. 2016;165(6):1332‐1345. doi:10.1016/j.cell.2016.05.041 27259147

[76] Heaney LM , Davies OG , Selby NM . Gut microbial metabolites as mediators of renal disease: do short‐chain fatty acids offer some hope? Futur Sci OA. 2019;5(4):FSO384. doi:10.4155/fsoa-2019-0013 PMC6511935 31114707

[77] Bongiovanni T , Yin MOL , Heaney LM . The athlete and gut microbiome: short‐chain fatty acids as potential ergogenic aids for exercise and training. Int J Sports Med. 2021;42(13):1143‐1158. doi:10.1055/a-1524-2095 34256388

[78] Nicholson JK , Wilson ID . Understanding “Global” systems biology: metabonomics and the continuum of metabolism. Nat Rev Drug Discov. 2003;2(8):668‐676. doi:10.1038/nrd1157 12904817

[79] Dunn WB , Broadhurst DI , Atherton HJ , Goodacre R , Griffin JL . Systems level studies of mammalian metabolomes: the roles of mass spectrometry and nuclear magnetic resonance spectroscopy. Chem Soc Rev. 2011;40(1):387‐426. doi:10.1039/B906712B 20717559

[80] Al‐Khelaifi F , Diboun I , Donati F , et al. A pilot study comparing the metabolic profiles of elite‐level athletes from different sporting disciplines. Sport Med ‐ Open. 2018;4(1):2. doi:10.1186/s40798-017-0114-z PMC5756230 29305667

[81] Heaney LM , Deighton K , Suzuki T . Non‐targeted metabolomics in sport and exercise science. J Sports Sci. 2019;37(9):959‐967. doi:10.1080/02640414.2017.1305122 28346122

[82] Li K , Naviaux JC , Bright AT , Wang L , Naviaux RK . A robust, single‐injection method for targeted, broad‐spectrum plasma metabolomics. Metabolomics. 2017;13(10):122. doi:10.1007/s11306-017-1264-1 28943831 PMC5583274

[83] Tso JV , Liu C , Turner CG , et al. Metabolic alterations differentiating cardiovascular maladaptation from athletic training in American‐style football athletes. Med Sci Sport Exerc. 2022;54(10):1617‐1624. doi:10.1249/MSS.0000000000002960 PMC9481654 35617604

[84] Henderson B , Meurs J , Lamers CR , et al. Non‐Invasive monitoring of inflammation in inflammatory bowel disease patients during prolonged exercise via exhaled breath volatile organic compounds. Metabolites. 2022;12(3):224. doi:10.3390/metabo12030224 35323667 PMC8948819

[85] Gehlert S , Weinisch P , Römisch‐Margl W , et al. Effects of acute and chronic resistance exercise on the skeletal muscle metabolome. Metabolites. 2022;12(5):445. doi:10.3390/metabo12050445 35629949 PMC9142957

[86] Wu L , Wang J , Cao X , Tian Y , Li J , Effect of acute high‐intensity exercise on myocardium metabolic profiles in rat and human study via metabolomics approach. *Sci Rep* . 2022;12(1):6791. doi:10.1038/s41598-022-10976-5 PMC9042871 35473956

[87] Nelson AB , Chow LS , Stagg DB , et al. Acute aerobic exercise reveals that FAHFAs distinguish the metabolomes of overweight and normal‐weight runners. JCI Insight. 2022;7(7):e158037. doi:10.1172/jci.insight.158037 35192550 PMC9057596

[88] Park J‐S , Kim Y‐J , Heo W , Kim S . The study of variation of metabolites by sleep deficiency, and intervention possibility of aerobic exercise. Int J Environ Res Public Health. 2022;19(5):2774. doi:10.3390/ijerph19052774 35270465 PMC8910362

[89] Zhao S , Chi A , Wan B , Liang J . differential metabolites and metabolic pathways involved in aerobic exercise improvement of chronic fatigue symptoms in adolescents based on gas chromatography–mass spectrometry. Int J Environ Res Public Health. 2022;19(4):2377. doi:10.3390/ijerph19042377 35206569 PMC8872503

[90] Zou L . Pivotal dominant bacteria ratio and metabolites related to healthy body index revealed by intestinal microbiome and metabolomics. Indian J Microbiol. 2022;62(1):130‐141. doi:10.1007/s12088-021-00989-5 35068612 PMC8758854

[91] Babu AF , Csader S , Männistö V , et al. Effects of exercise on NAFLD using non‐targeted metabolomics in adipose tissue, plasma, urine, and stool. Sci Rep. 2022;12(1):6485. doi:10.1038/s41598-022-10481-9 35444259 PMC9019539

[92] Byerley LO , Gallivan KM , Christopher CJ , et al. Gut microbiome and metabolome variations in self‐identified muscle builders who report using protein supplements. Nutrients. 2022;14(3):533. doi:10.3390/nu14030533 35276896 PMC8839395

[93] Germain A , Giloteaux L , Moore GE , et al. Plasma metabolomics reveals disrupted response and recovery following maximal exercise in myalgic encephalomyelitis/chronic fatigue syndrome. JCI Insight. 2022;7(9):e157621. doi:10.1172/jci.insight.157621 35358096 PMC9090259

[94] Heaney LM , Kang S , Turner MA , Lindley MR , Thomas CLP . The impact of a graded maximal exercise protocol on exhaled volatile organic compounds: a pilot study. Molecules. 2022;27(2):370. doi:10.3390/molecules27020370 35056684 PMC8779231

[95] Paparo L , Maglio MA , Cortese M , et al. A new butyrate releaser exerts a protective action against SARS‐CoV‐2 infection in human intestine. Molecules. 2022;27(3):862. doi:10.3390/molecules27030862 35164139 PMC8838168

[96] Savikj M , Stocks B , Sato S , et al. Exercise timing influences multi‐tissue metabolome and skeletal muscle proteome profiles in type 2 diabetic patients – A randomized crossover trial. Metabolism. 2022;135:155268. doi:10.1016/j.metabol.2022.155268 35908579

[97] Tarkhan AH , Anwardeen NR , Sellami M , et al. Comparing metabolic profiles between female endurance athletes and non‐athletes reveals differences in androgen and corticosteroid levels. J Steroid Biochem Mol Biol. 2022;219:106081. doi:10.1016/j.jsbmb.2022.106081 35182726

[98] Piper T , Geyer H , Haenelt N , Huelsemann F , Schaenzer W , Thevis M . Current insights into the steroidal module of the athlete biological passport. Int J Sports Med. 2021;42(10):863‐878. doi:10.1055/a-1481-8683 34049412 PMC8445669

[99] Statuta SM , Asif IM , Drezner JA . Relative energy deficiency in sport (RED‐S). Br J Sports Med. 2017;51(21):1570‐1571. doi:10.1136/bjsports-2017-097700 28684389

[100] Dipla K , Kraemer RR , Constantini NW , Hackney AC . Relative energy deficiency in sports (RED‐S): elucidation of endocrine changes affecting the health of males and females. Hormones. 2021;20(1):35‐47. doi:10.1007/s42000-020-00214-w 32557402

[101] Sumner LW , Amberg A , Barrett D , et al. Proposed minimum reporting standards for chemical analysis. Metabolomics. 2007;3(3):211‐221. 24039616 10.1007/s11306-007-0082-2 PMC3772505

[102] Latino F , Cataldi S , Carvutto R , et al. The importance of lipidomic approach for mapping and exploring the molecular networks underlying physical exercise: a systematic review. Int J Mol Sci. 2021;22(16):8734. doi:10.3390/ijms22168734 34445440 PMC8395903

[103] Hussan H , Abu Dayyeh BK , Chen J , et al. Adjustable intragastric balloon leads to significant improvement in obesity‐related lipidome and fecal microbiome profiles: a proof‐of‐concept study. Clin Transl Gastroenterol. 2022;13(7):e00508. doi:10.14309/ctg.0000000000000508 35905412 PMC10476793

[104] San Martin R , Brandao CFC , Junqueira‐Franco MVM , et al. Untargeted lipidomic analysis of plasma from obese women submitted to combined physical exercise. Sci Rep. 2022;12(1):11541. doi:10.1038/s41598-022-15236-0 35798803 PMC9263166

[105] Bowman ER , Wilson M , Riedl KM , et al. Lipidome alterations with exercise among people with and without HIV: an exploratory study. AIDS Res Hum Retroviruses. 2022;38(7):544‐551. doi:10.1089/aid.2021.0154 35302400 PMC9297322

[106] Arthur KL , Turner MA , Reynolds JC , Creaser CS . Increasing peak capacity in nontargeted omics applications by combining full scan field asymmetric waveform ion mobility spectrometry with liquid chromatography‐mass spectrometry. Anal Chem. 2017;89(6):3452‐3459. doi:10.1021/acs.analchem.6b04315 28230966

[107] Vvedenskaya O , Holčapek M , Vogeser M , Ekroos K , Meikle PJ , Bendt AK . Clinical lipidomics – A community‐driven roadmap to translate research into clinical applications. J Mass Spectrom Adv Clin Lab. 2022;24:1‐4. doi:10.1016/j.jmsacl.2022.02.002 35199094 PMC8844780

[108] McDonald JG , Ejsing CS , Kopczynski D , et al. Introducing the lipidomics minimal reporting checklist. Nat Metab. 2022;4(9):1086‐1088. doi:10.1038/s42255-022-00628-3 35934691

[109] Hittel DS , Hathout Y , Hoffman EP . Proteomics and systems biology in exercise and sport sciences research. Exerc Sport Sci Rev. 2007;35(1):5‐11. doi:10.1097/jes.0b013e31802d744a 17211187

[110] Zhang Y , Fonslow BR , Shan B , Baek M‐C , Yates JR . Protein analysis by shotgun/bottom‐up proteomics. Chem Rev. 2013;113(4):2343‐2394. doi:10.1021/cr3003533 23438204 PMC3751594

[111] Taylor CF , Paton NW , Lilley KS , et al. The minimum information about a proteomics experiment (MIAPE). Nat Biotechnol. 2007;25(8):887‐893. doi:10.1038/nbt1329 17687369

[112] Coudy‐Gandilhon C , Gueugneau M , Chambon C , et al. A single bout of ultra‐endurance exercise reveals early signs of muscle aging in master athletes. Int J Mol Sci. 2022;23(7):3713. doi:10.3390/ijms23073713 35409073 PMC8998696

[113] Dearlove DJ , Hauton D , et al. The effects of endogenously‐ and exogenously‐induced hyperketonemia on exercise performance and adaptation. Physiol Rep. 2022;10(10):e15309. doi:10.14814/phy2.15309 35614576 PMC9133544

[114] Vanderboom P , Zhang X , Hart CR , et al. Impact of obesity on the molecular response to a single bout of exercise in a preliminary human cohort. Obesity. 2022;30(5):1091‐1104. doi:10.1002/oby.23419 35470975 PMC9048146

[115] Deane CS , Phillips BE , Willis CRG , et al. Proteomic features of skeletal muscle adaptation to resistance exercise training as a function of age. GeroScience. doi:10.1007/s11357-022-00658-5. Published online 2022. PMC10400508 36161583

[116] Wåhlén K , Yan H , Welinder C , et al. Proteomic investigation in plasma from women with fibromyalgia in response to a 15‐wk resistance exercise intervention. Med Sci Sports Exerc. 2022;54(2):232‐246. doi:10.1249/MSS.0000000000002790 35029590 PMC8754090

[117] Militello R , Pinto G , Illiano A , et al. Modulation of plasma proteomic profile by regular training in male and female basketball players: a preliminary study. Front Physiol. 2022;13:813447. doi:10.3389/fphys.2022.813447 35360242 PMC8964093

[118] Pacheco VB , Nery G , Fernandes LL , et al. Salivary proteome, inflammatory, and netosis biomarkers in older adult practitioners and nonpractitioners of physical exercise. González A. Oxid Med Cell Longev. 2022;2022:3725056. doi:10.1155/2022/3725056 35502212 PMC9056209

[119] Ventura TMO , Santos KO , Braga AS , et al. Salivary proteomic profile of young adults before and after the practice of interval exercise: preliminary results. Sport Sci Health. 2022;18(3):983‐997. doi:10.1007/s11332-021-00883-z

[120] Daisy CC , Varinos S , Howell DR , et al. Proteomic discovery of noninvasive biomarkers associated with sport‐related concussions. Neurology. 2022;98(2):e186‐e198. doi:10.1212/WNL.0000000000013001 34675105 PMC8762586

[121] Cartee GD , Hepple RT , Bamman MM , Zierath JR . Exercise promotes healthy aging of skeletal muscle. Cell Metab. 2016;23(6):1034‐1047. doi:10.1016/j.cmet.2016.05.007 27304505 PMC5045036

[122] Wilkinson DJ , Brook MS , Smith K , Atherton PJ . Stable isotope tracers and exercise physiology: past, present and future. J Physiol. 2017;595(9):2873‐2882. doi:10.1113/JP272277 27610950 PMC5407962

[123] Piper T , Emery C , Saugy M . Recent developments in the use of isotope ratio mass spectrometry in sports drug testing. Anal Bioanal Chem. 2011;401(2):433‐447. doi:10.1007/s00216-011-4886-6 21448602

[124] Alvarez‐Jimenez L , Moreno‐Cabañas A , Morales‐Palomo F , Mora‐Rodriguez R . Effects of metabolic syndrome on fuel utilization during exercise in middle‐aged moderately trained individuals. J Appl Physiol. 2022;132(6):1423‐1431. doi:10.1152/japplphysiol.00040.2022 35511719

[125] Hearris MA , Pugh JN , Langan‐Evans C , et al. 13 C‐glucose‐fructose labeling reveals comparable exogenous CHO oxidation during exercise when consuming 120 g/h in fluid, gel, jelly chew, or coingestion. J Appl Physiol. 2022;132(6):1394‐1406. doi:10.1152/japplphysiol.00091.2022 35446596

[126] Rowe JT , King RFGJ , King AJ , et al. Glucose and fructose hydrogel enhances running performance, exogenous carbohydrate oxidation, and gastrointestinal tolerance. Med Sci Sports Exerc. 2022;54(1):129‐140. doi:10.1249/MSS.0000000000002764 34334720

[127] Brennan AM , Standley RA , Anthony SJ , et al. Weight loss and exercise differentially affect insulin sensitivity, body composition, cardiorespiratory fitness, and muscle strength in older adults with obesity: a randomized controlled trial. Journals Gerontol Ser A. 2022;77(5):1088‐1097. doi:10.1093/gerona/glab240. Lipsitz LA. PMC9071425 34406407

[128] Podlogar T , Bokal Š , Cirnski S , Wallis GA . Increased exogenous but unaltered endogenous carbohydrate oxidation with combined fructose‐maltodextrin ingested at 120 g h−1 versus 90 g h−1 at different ratios. Eur J Appl Physiol. 2022;122(11):2393‐2401. doi:10.1007/s00421-022-05019-w 35951130 PMC9560939

[129] Crossland H , Brook MS , Quinlan JI , et al. Metabolic and molecular responses of human patellar tendon to concentric‐ and eccentric‐type exercise in youth and older age. GeroScience. doi:10.1007/s11357-022-00636-x. Published online 2022. PMC9886711 35948859

[130] Davies RW , Kozior M , Lynch AE , et al. The Effect of Fava Bean (Vicia faba L.) protein ingestion on myofibrillar protein synthesis at rest and after resistance exercise in healthy, young men and women: a randomised control trial. Nutrients. 2022;14(18):3688. doi:10.3390/nu14183688 36145064 PMC9502734

[131] Mazzulla M , Hodson N , West DWD , Kumbhare DA , Moore DR . A non‐invasive13 CO2 breath test detects differences in anabolic sensitivity with feeding and heavy resistance exercise in healthy young males: a randomized control trial. Appl Physiol Nutr Metab. 2022;47(8):860‐870. doi:10.1139/apnm-2021-0808 35609328

[132] Gharahdaghi N , Rudrappa S , Brook MS , et al. Pharmacological hypogonadism impairs molecular transducers of exercise‐induced muscle growth in humans. J Cachexia Sarcopenia Muscle. 2022;13(2):1134‐1150. doi:10.1002/jcsm.12843 35233984 PMC8977972

[133] Barney DE , Ippolito JR , Berryman CE , Hennigar SR . A prolonged bout of running increases hepcidin and decreases dietary iron absorption in trained female and male runners. J Nutr. 2022;152(9):2039‐2047. doi:10.1093/jn/nxac129 35661896

[134] Toro‐Román V , Siquier‐Coll J , Bartolomé I , Grijota FJ , Muñoz D , Maynar‐Mariño M . Influence of physical training on intracellular and extracellular zinc concentrations. J Int Soc Sports Nutr. 2022;19(1):110‐125. doi:10.1080/15502783.2022.2054665 35599919 PMC9116397

[135] Toro‐Román V , Bartolomé I , Siquier‐Coll J , MC Robles‐Gil , Muñoz D , Maynar‐Mariño M . Analysis of intracellular and extracellular selenium concentrations: differences according to training level. Nutrients. 2022;14(9):1857. doi:10.3390/nu14091857 35565824 PMC9102273

[136] Carter SJ , Singh H , Nabhan DC , Long EB , Hunter GR . Relative leg press strength relates to activity energy expenditure in older women: implications for exercise prescription. Exp Gerontol. 2022;169:111956. doi:10.1016/j.exger.2022.111956 36126803

[137] Cauci S , Tavano M , Curcio F , Francescato MP . Biomonitoring of urinary metals in athletes according to particulate matter air pollution before and after exercise. Environ Sci Pollut Res. 2022;29(18):26371‐26384. doi:10.1007/s11356-021-17730-w PMC8637506 34855175

[138] Logue DM , Madigan SM , Melin A , et al. Low energy availability in athletes 2020: an updated narrative review of prevalence, risk, within‐day energy balance, knowledge, and impact on sports performance. Nutrients. 2020;12(3):835. doi:10.3390/nu12030835 32245088 PMC7146210

[139] FDA . Bioanalytical method validation guidance for industry. Published 2018. Accessed January 24, 2022. https://www.fda.gov/files/drugs/published/Bioanalytical‐Method‐Validation‐Guidance‐for‐Industry.pdf

[140] EMA . Guideline on bioanalytical method validation. Published 2011. Accessed January 24, 2022. https://www.ema.europa.eu/en/documents/scientific‐guideline/guideline‐bioanalytical‐method‐validation_en.pdf

[141] Paul RW , Sonnier JH , Johnson EE , et al. Inequalities in the evaluation of male versus female athletes in sports medicine research: a systematic review. Am J Sports Med. doi:10.1177/03635465221131281. Published online 2022. 36453705

[142] Costello JT , Bieuzen F , Bleakley CM . Where are all the female participants in sports and exercise medicine research? Eur J Sport Sci. 2014;14(8):847‐851. doi:10.1080/17461391.2014.911354 24766579

[143] Pellegrino JK , Anthony TG , Gillies P , Arent SM . The exercise metabolome: acute aerobic and anaerobic signatures. J Int Soc Sports Nutr. 2022;19(1):603‐622. doi:10.1080/15502783.2022.2115858 36250148 PMC9559054

[144] Janse de Jonge XAK. Effects of the menstrual cycle on exercise performance. Sport Med. 2003;33(11):833‐851. doi:10.2165/00007256-200333110-00004 12959622

[145] Krasowski MD , Drees D , Morris CS , Maakestad J , Blau JL , Ekins S . Cross‐reactivity of steroid hormone immunoassays: clinical significance and two‐dimensional molecular similarity prediction. BMC Clin Pathol. 2014;14(1):33. doi:10.1186/1472-6890-14-33 25071417 PMC4112981

[146] Taylor AE , Keevil B , Huhtaniemi IT . Mass spectrometry and immunoassay: how to measure steroid hormones today and tomorrow. Eur J Endocrinol. 2015;173(2):D1‐D12. doi:10.1530/EJE-15-0338 25877990

[147] Monaghan PJ , Keevil BG , Stewart PM , Trainer PJ . Case for the wider adoption of mass spectrometry‐based adrenal steroid testing, and beyond. J Clin Endocrinol Metab. 2014;99(12):4434‐4437. doi:10.1210/jc.2014-2258 25322267

[148] Burr JF , Cheung CP , Kasper AM , Gillham SH , Close GL . Cannabis and athletic performance. Sport Med. 2021;51(S1):75‐87. doi:10.1007/s40279-021-01505-x PMC8566388 34515970

[149] Rojas‐Valverde D . Potential role of cannabidiol on sports recovery: a narrative review. Front Physiol. 2021;12:722550. doi:10.3389/fphys.2021.722550 34413793 PMC8369499

[150] Mareck U , Fusshöller G , Geyer H , Huestis MA , Scheiff AB , Thevis M . Preliminary data on the potential for unintentional antidoping rule violations by permitted cannabidiol (CBD) use. Drug Test Anal. 2021;13(3):539‐549. doi:10.1002/dta.2959 33125823

