# Supplementary material for: Mass Sportrometry: An annual look back at applications of mass spectrometry in sport and exercise science
Source: Anal Sci Adv. 2023 Mar 31;4(3-4):60–80. doi: 10.1002/ansa.202300003 (PMC10989560; doi:10.1002/ansa.202300003)
Supplement: Supplementary file 1 — Supporting Information [file ANSA-4-60-s001.pdf]

SUPPLEMENTARY MATERIAL

Mass Sportrometry: An Annual Look Back at Applications of Mass Spectrometry in Sport and Exercise Science

Marilyn LY Ong[1,2], Christopher G Green[1], Samantha N Rowland[1], Liam M Heaney[1*]

[1] School of Sport, Exercise & Health Sciences, Loughborough University, Loughborough, UK

[2] Exercise and Sports Science Programme, School of Health Sciences, Universiti Sains Malaysia, Kota Bharu, Kelantan, Malaysia

Corresponding Author:

[*] Dr Liam M Heaney, School of Sport, Exercise & Health Sciences, Loughborough University, Loughborough, UK. Email: L.M.Heaney2@Lboro.ac.uk

Table S1. Studies applying a targeted mass spectrometry approach to human sports science and exercise research in 2022.

Reference	Subjects	N	Exercise Type	Analytical Technique	Targets	Matrix	50%+ Female Participants?
**Allman BR et al**	Sedentary pregnant women with obesity	80	Aerobic and resistance	LC-MS	Long-chain acylcarnitine	Serum	Y
**Bekesiene S et al**	Military soldiers at compulsory military conscripts	112	Military training	LC-MS	Cortisol, cortisone, DHEA	Hair	
**Bernhardt V et al**	Women with obesity	17	Submaximal, constant-load cycling	Direct MS	Gas fractions	Breath	Y
**Bjørnebekk A et al**	Male weightlifter	141	Strength training	GC-MS and LC-MS	Androgen	Urine	
**Carswell AT et al**	British Army trainee recruits – Infantry	1,527	Army training	LC-MS	Vitamin D metabolites: 25(OH)D and 24,25(OH)2D	Serum	
**Chaouachi M et al**	Elite male rugby union players	17	Repeated high-intensity exercise to exhaustion	LC-MS	F2α-isoprostanes	Plasma	
**Dalgaard LB et al**	Untrained, menopausal women	32	Resistance training	LC-MS	Estradiol, testosterone hormone	Serum	Y
**Eugster PJ et al**	Healthy, non-smoker males	6	Moderate cycling	LC-MS	NPYs and CATs	Plasma	
**Forteza F et al**	Active women with no underlying metabolic conditions	7	Maximal running	LC-MS	NAEs, MAGs, PUFAs	Plasma	Y
**Gagesch M et al**	Healthy European adults aged 70 years or older	1,137	Strength and flexibility exercise, regular/chronic exercise	LC-MS	25(OH)D	Serum	Y

**Gillies NA et al**	Sedentary elderly	95	Resistance training	LC-MS	Betaine, choline, DMG, SAH, SAM; and amino acids: cysteine, cystathionine, glycine, homocysteine, methionine, serine	Plasma	Y
**Grillet P et al**	Patients diagnosed with muscle disease (myopathy)	19	Cardiopulmonary exercise test	LC-MS	Lactate, pyruvate and ketone bodies	Plasma	Y
**Güntner AT et al**	Type 1 diabetes volunteers	19	Moderate exercise	PTR-TOF-MS	Acetone, ethanol, isoprene	Breath	Y
**Jaramillo-Morales et al**	Stage 3 and 4 chronic kidney disease patients	92	Aerobic exercise and caloric restriction	GC-MS	F2-isoprostane and isofuran	Plasma	
**Jung C et al**	Individuals with non-alcoholic fatty liver disease	268,946	Self-reported exercise sessions per week	LC-MS	Creatinine	Serum	Y
**Jurado-Fasoli L et al**	Middle-aged, sedentary adults	65	High-intensity interval training	LC-MS	Oxylipins, eCBs, and eCBs-like molecules	Plasma	Y
**Kosaki K et al**	Trained, recreational marathon runners	23	Endurance, marathon	LC-MS	Hypoxanthine, xanthine, uric acid ($^{3}C_2$, $^{15}N_2$)	Plasma	
**Kunz HE et al**	Sedentary, older adults	63	Acute, high-intensity resistance leg extension exercise	LC-MS	EPA and DHA	Red blood cells	Y
**Lake SL et al**	Aged, sedentary men and women	203	Low to moderate progressive intensity aerobic exercise	Direct MS	Gas fractions	Breath	Y
**Legaard GE et al**	Persons diagnosed with type 2 diabetes <10 years	77	High-volume, moderate-to-vigorous aerobic exercise combined with resistance exercise	LC-MS	8-oxoGuo and 8-oxodG	Urine	Y

**Li K et al**	Physically active, healthy, young adults	19	Acute endurance exercise	LC-MS	Intra- and extracellular metabolites	Plasma, CSF	
**Li VL et al**	Healthy individuals	36	Acute, endurance running	LC-MS	N-lactoyl-phenylalanine	Plasma	
**Li X et al**	Males with methamphetamine dependence	75	Aerobic exercise	LC-MS	Metabolites related to methamphetamine	Serum	
**Liu S et al**	Older adults	66	Six-minute walk and repeated leg muscle isometric contractions until exhaustion	LC-MS	Urolithin A and urolithin A glucuronide	Plasma	Y
**Løkken N et al**	Patients with glycogen storage disease type V	12	Moderate-high cycling	LC-MS	Pyruvate, lactate, and Acetoacetate	Plasma	Y
**Luk AWS et al**	Competitive bodybuilders	5	Bodybuilding	LC-MS	Amino acids, nucleotides, neurotransmitters and vitamins, nucleoside phosphates, high-energy intermediates, organic acids, Krebs cycle intermediates, and glycolytic intermediates	Serum	
**Maalouf NM et al**	Physically active, elderly men with advanced prostate cancer	16	Free-living physical activity	LC-MS	Testosterone, estradiol	Serum	
**Machek SB et al**	Recreationally resistance-trained men	5	No intervention, cross-sectional	LC-MS	Betaine	Serum	
**Moriyama S et al**	Healthy young men and women	13	Acute knee extension exercise	Direct MS	Gas fractions	Breath	
**Nishimura Y et al**	Moderately trained, healthy, young males	10	Whole-body resistance exercise	LC-MS	D5- and 15N-phenylalanine, amino acids	Serum, muscle	

**Patten RK et al**	Inactive, overweight women diagnosed with polycystic ovary syndrome	29	Moderate-intensity continuous training and high-intensity interval training	LC-MS	Total testosterone, free testosterone, dihydrotestosterone, estradiol, androstenedione, total cholesterol, triglycerides, high-density lipoprotein cholesterol, low-density lipoprotein cholesterol	Serum	Y
**Perreault et al**	Healthy pregnant women	187	Walking	LC-MS	Vitamin D metabolites: 25(OH)D and 1,25(OH)2D	Serum	Y
**Puigarnau S et al**	Trail runners	33	Aerobic, endurance running	LC-MS	Metabolites related to amino acid, purine, and nitrogen metabolism	Capillary blood	
**Revuelta Iniesta R et al**	Children and adults with cystic fibrosis	90	Cardiopulmonary exercise testing	LC-MS	25(OH)D	Plasma	
**Rodas G et al**	Professional football player	28	Intermittent, football season training monitoring	LC-MS	Amino acids and metabolites related to tryptophan and phenylalanine pathways	Urine	Y
**Sahinovic A et al**	Endurance-trained males	9	Submaximal followed by incremental running to volitional exhaustion	LC-MS	Cannabidiol	Plasma	
**Singh A et al**	Aged, sedentary and overweight adults	79	Incremental submaximal cycling	LC-MS, GC-MS	Urolithin A	Plasma	Y
**Strom CJ et al**	Pregnant women	30	Moderate intensity exercise	LC-MS	DHA and EPA	Plasma	Y

**Tarumi T et al**	Cognitively normal older adults	73	Aerobic exercise training or active control group: stretching-and-toning	Direct MS	Gas fractions	Exhaled breath	Y
**Tataka Y et al**	Young, active men	16	Acute, prolonged strenuous exercise	LC-MS	Lactulose and mannitol	Plasma	
**Thams L et al**	Children	183	No exercise, fitness assessment	LC-MS	Vitamin D metabolites: 25(OH)D2 and 25(OH)D3	Serum	Y
**Tomoto T et al**	Cognitively normal older adults	73	Aerobic exercise training or active control group: stretching-and-toning	Direct MS	Gas fractions	Exhaled breath	Y
**Torquati L et al**	Type 2 diabetic men and women	14	Combined aerobic and resistance moderate-intensity continuous or combined aerobic and resistance high-intensity interval training	GC-MS	SCFAs	Feces	Y
**Tsikas D et al**	Healthy, young men	17	Intermittent, high-intensity and high-volume cycling	GC-MS	Nitrate, nitrite, amino acids, and their metabolites (including homoarginine and guanidinoacetate)	Plasma	
**Wang R et al**	Professional table tennis players	29	Muscle strength and aerobic endurance exercise	LC-MS	Metabolome related to β-glucan	Plasma	
**Wangdi JT et al**	Recreationally, active male	10	Muscle-damaging exercise protocol (eccentric knee flexion contractions)	LC-MS	Phenolics: Protocatechuic acid, 4-hydroxybenzoic acid, hippuric acid, vanillic acid, ferulic acid, and isoferulic acid	Plasma	

**Warensjö Lemming E et al**	School-going Riksmaten adolescents (from 2016-17 Swedish National Survey)	1,100	Free-living physical activity	LC-MS	25(OH)D, 25(OH)D3 and 25(OH)D2	Plasma	Y
**Withycombe JS et al**	Children with cancer (survivors and still receiving therapy)	32	Daily physical activity	LC-MS	Metabolites related to energy expenditure	Urine	
**Williams CJ et al**	Healthy inactive adults	40	Supervised HIIT training sessions	GC-MS	SCFAs	Fecal	Y
**Xu Y et al**	First-year university students	200	Multiple sports	LC-MS	Dimethoate, BaP and BPA	Urine	Y
**Zeng G et al**	Adolescents from 2003-2004 National Health and Nutrition Examination Survey	491	Free-living physical activity	LC-MS	PFASs	Serum	
**Zhang Q et al**	Elite athlete	54	No exercise. Elite athlete cohort (cross-sectional)	GC-MS	Total fatty acids	Serum	Y

Abbreviations: MS – mass spectrometry; LC – liquid chromatography; GC – gas chromatography; PTR – proton transfer reaction; TOF – time-of-flight; DHEA – Dehydroepiandrosterone; Dehydroepiandrosterone sulfate – DHEAs; DHT – dihydrotestosterone; 17-OHP – 17-OH-progesterone; NPYs – neuropeptides; CATs – catecholamines; 25(OH)D – 25-hydroxyvitamin D; 24,25(OH)2D – 24,25-dihydroxyvitamin D; DMG – dimethylglycine; SAH – S-adenosylhomocysteine; SAM – S-adenosylmethionine; EPA– eicosapentaenoic acid; DHA – docosahexaenoic acid; 8-oxoGuo – 8-oxo-7,8-dihydroguanosine; and 8-oxodG – 8-oxo-7,8-dihydro-2 ′-deoxyguanosine; NAEs – N-acyl-ethanolamines, MAGs – 2-monoacylglycerols; PUFAs – n-3 polyunsaturated fatty acids; SCFAs – short-chain fatty acids; BaP – benzo(a)pyrene; BPA – bisphenol A; PFASs – perfluoroalkyl substances

Table S2. Studies applying a metabolomics-based mass spectrometry approach to human sports science and exercise research in 2022.

Reference	Subjects	N	Exercise Type	Analytical Technique	Matrix	50%+ Female Participants?
**Babu AF et al**	Patients with non-alcoholic fatty liver disease	46	High-intensity interval training	LC-MS	Plasma, Adipose tissue, urine and stool	Y
**Byerley LO et al**	Online sports & Health Science majors students and self-identified as muscle builders	22	Self-reported resistance training	LC-MS	Fecal	
**Gehlert S et al**	Moderately, resistance-trained males	14	Acute and chronic leg resistance exercise	LC-MS	Vastus lateralis muscle	
**Germain A et al**	Myalgic Encephalomyelitis/ chronic fatigue syndrome patients	105	Maximal cycling exercise	LC-MS	Plasma	Y
**Heaney LM et al**	Healthy, active males	33	Maximal exercise	TD-GC-MS	Breath	
**Henderson B et al**	Inflammatory bowel disease patients	37	Repeated and prolonged moderate-intensity exercise	PTR-TOF-MS	Breath	Y
**Li VL et al**	Healthy individuals	36	Acute, endurance running	LC-MS	Plasma	
**Liu S et al**	Older adults	66	Six-minute walk and repeated leg muscle isometric contractions until exhaustion	LC-MS	Plasma	Y
**Nelson AB et al**	Trained runners	25	Acute, prolonged moderate-intensity running	LC-MS	Serum	Y
**Park J et al**	Healthy university students with normal BMI	32	Low, moderate and high aerobic exercises	GC-MS	Serum	
**Pellegrino JK et al**	Healthy recreationally active adults	40	Combined (aerobic and resistance)	LC-MS	Serum	Y
**Savikj M et al**	Type 2 diabetic men	8	Cycling HIT	LC-MS	Serum	

**Singh A et al**	Aged, sedentary and overweight adults	79	Incremental submaximal cycling, muscle strength and endurance tests	LC-MS, GC-MS	Plasma	Y
**Tarkhan AH et al**	Elite female endurance athletes	51	Elite sports - athletics, boxing, rowing, cycling, kayaking, swimming, hockey, tennis	LC-MS	Serum	Y
**Tso JV et al**	Collegiate freshman American-style footballers	82	No exercise intervention; Longitudinal cohort	LC-MS	Plasma	
**Wu L et al**	Healthy males from Physical Education Institute	8	Acute, high-intensity intermittent cycling	LC-MS	Serum	
**Zhao S et al**	Male, high school students with chronic fatigue syndrome	46	Aerobic exercise	GC-MS	Urine	
**Zou L et al**	Healthy amateur marathon runners (adults)	20	No exercise intervention but sampled from amateur runners (parallel to other studies)	LC-MS	Fecal	

Abbreviations: LC – Liquid chromatography; MS – mass spectrometry; TD – thermal desorption; GC – gas chromatography; PTR – proton transfer reaction; TOF – Time-of-flight; VOCs - Volatile Organic Compounds; eCBs – endocannabinoids; CSF – cerebrospinal fluids

Table S3. Studies applying a lipidomics-based mass spectrometry approach to human sports science and exercise research in 2022.

Reference	Subjects	N	Exercise Type	Analytical Technique	Matrix	50%+ Female Participants?
**Bowman ER et al**	Aged, sedentary HIV patients	49	Combined aerobic and resistance	LC-MS	Serum	
**Hussan H et al**	Obese patients with intragastric balloons	12	Moderate aerobic and resistance	LC-MS	Serum	Y
**Nelson AB et al**	Trained runners	25	Acute, prolonged moderate-intensity running	LC-MS	Serum	Y
**San Martin R et al**	Sedentary obese adult females	14	Combined aerobic and resistance	LC-MS	Plasma	Y

Abbreviations: HIV – human immunodeficiency virus; LC – Liquid chromatography; MS – mass spectrometry

Table S4. Studies applying a proteomics-based mass spectrometry approach to human sports science and exercise research in 2022.

Reference	Subjects	N	Exercise Type	Analytical Technique	Matrix	50%+ Female Participants?
**Coudy-Gandilhon C et al**	Highly trained masters athletes	11	Ultra-endurance running	LC-MS	Vastus lateralis muscle	
**Daisy CC et al**	Contact and non-contact sport collegiate athletes with concussion	95	Single-task gait velocity (functional)	LC-MS	Urine	Y
**Deane CS et al**	Young, recreationally active males and females	16	Whole-body resistance exercise training	LC-MS	m. vastus lateralis	
**Dearlove DJ et al**	Trained, endurance athletes	21	Endurance race/simulated in laboratory	LC-MS	Skeletal muscle	
**Militello R et al**	Male and female professional basketball players	40	In-season training period/non-competition	LC-MS	Plasma	Y
**Pacheco VB et al**	Older adults practitioners of physical exercise	10	Resistance and aerobic training	LC-MS	Saliva	
**Savikj M et al**	Type 2 diabetic men	8	Cycling HIT	LC-MS	Vastus lateralis muscle	
**Singh A et al**	Aged, sedentary and overweight adults	79	Incremental submaximal cycling, muscle strength and endurance tests	LC-MS	Vastus lateralis	Y
**Vanderboom P et al**	Obese adults	8	Acute, short-term, high intensity single-leg cycling	LC-MS	Vastus lateralis muscle	Y
**Ventura TMO et al**	Healthy young adults	5	Interval exercise	LC-MS	Saliva	
**Wahlen K et al**	Women with fibromyalgia and healthy controls	45	Resistance exercise	LC-MS	Plasma	Y

Abbreviations: LC – liquid chromatography, MS – mass spectrometry;

Table S5. Studies applying an isotope ratio and elemental mass spectrometry approach to human sports science and exercise research in 2022.

Reference	Subjects	N	Exercise Type	Analytical Technique	Targets	Matrix	50%+ Female Participants?
**Alvarez-Jimenez L et al**	Middle-aged, moderately trained individuals with metabolic syndrome	17	Moderate-to-high cycling intensities	GC-MS, IRMS	Glucose, glycerol	Red blood cells	
**Barney DE et al**	Collegiate cross-country runners	28	Endurance, prolonged running	ICP-MS	Stable iron isotopes	Red blood cells	Y
**Brennan AM et al**	Older adults with obesity	61	Moderate walking exercise (aerobic)	IRMS	Glucose [6,6-$^{2}H_2$]	*Not mentioned*	Y
**Carter SJ et al**	Untrained older women	76	Functional, strength training	IRMS	^{2}H and ^{18}O	Urine	Y
**Cauci S et al**	Non-professional male American football players	8	Training sessions consisted of agility drills, technical exercises and simulated football match	ICP-MS	15 types of metals related to air pollution	Urine	
**Crossland H et al**	Healthy, recreationally active young and older men	54	Eccentric and concentric exercise training	GC-IRMS	Protein-bound alanine (hydroxyproline/ proline ratio)	Tendon	
**Davies RW et al**	Young, healthy recreationally active adults	16	Resistance training	GC- IRMS	Myofibrillar protein-bound alanine	m. vastus lateralis muscle	
**Dearlove DJ et al**	Trained, endurance athletes	21	Endurance race/simulated in laboratory	GC-IRMS	$^{13}CO_2$	Breath	
**Gharahdaghi N et al**	Non-hypogonadal men	16	Whole-body resistance exercise	IRMS	Myofibrillar protein-bound alanine	Vastus lateralis muscle	
**Hearris MA et al**	Amateur, endurance-trained male cyclists	9	Cycling endurance exercise	GC-IRMS	$^{13}C/^{12}C$	Breath CO_2	

**Kunz HE et al**	Sedentary, older adults	63	Acute, high-intensity resistance leg extension exercise	GC-IRMS	Whole-body protein turnover: $^{13}C_6$ -phenylalanine, $^{13}C_6$-tyrosine, and ^{15}N-tyrosine; Skeletal muscle fractional synthesis rates: $^{13}C_6$-phenylalanine	Plasma, vastus lateralis muscle	Y
**Løkken N et al**	Patients with glycogen storage disease type V	12	Moderate-high cycling	GC-IRMS	Hydroxybutyrate, glucose, and glycerol	Plasma, breath	Y
**Mazzulla et al**	Recreationally active males	22	Whole-body resistance exercise	GC-MS, IRMS	Leucine, $^{13}CO_2$	Plasma, breath	
**Nishimura Y et al**	Moderately trained, healthy males	10	Whole-body resistance exercise	IRMS	D_5- and ^{15}N-phenylalanine, amino acids	Serum, muscle	
**Podlogar T et al**	Trained male cyclists	8	Prolonged, high-intensity cycling	GC-IRMS	^{13}C	Breath	
**Rowe JT et al**	Trained male endurance runners	11	Marathon, endurance	LC-IRMS	$^{13}C/^{12}C$ Glucose	Plasma	
**Toro-Román V et al**	Young male semi-professional soccer players	40	Physical performance test (Treadmill maximal graded test)	ICP-MS	Selenium	Plasma, serum, urine, erythrocytes and platelets	
**Toro-Román V et al**	Young male semi-professional soccer players	40	Physical performance test (Treadmill maximal graded test)	ICP-MS	Zinc	Erythrocyte, platelet, plasma, serum, and urine	

Abbreviations: GC – gas chromatography; MS – mass spectrometry; IR – isotope ratio; ICP – inductively coupled plasma; LC – liquid chromatography
